# OCEAN-C: mapping hubs of open chromatin interactions across the genome reveals gene regulatory networks

**DOI:** 10.1186/s13059-018-1430-4

**Published:** 2018-04-24

**Authors:** Tingting Li, Lumeng Jia, Yong Cao, Qing Chen, Cheng Li

**Affiliations:** 1 Peking-Tsinghua Center for Life Sciences, Academy for Advanced Interdisciplinary Studies; School of Life Sciences, Peking University, Beijing, 100871 China; 2 State Key Laboratory of Proteomics, National Center of Biomedical Analysis, Institute of Basic Medical Sciences, Beijing, 100850 China; 3 Center for Statistical Science; Center for Bioinformatics, Peking University, Beijing, 100871 China

## Abstract

We develop a method called open chromatin enrichment and network Hi-C (OCEAN-C) for antibody-independent mapping of global open chromatin interactions. By integrating FAIRE-seq and Hi-C, OCEAN-C detects open chromatin interactions enriched by active *cis*-regulatory elements. We identify more than 10,000 hubs of open chromatin interactions (HOCIs) in human cells, which are mainly active promoters and enhancers bound by many DNA-binding proteins and form interaction networks crucial for gene transcription. In addition to identifying large-scale topological structures, including topologically associated domains and A/B compartments, OCEAN-C can detect HOCI-mediated chromatin interactions that are strongly associated with gene expression, super-enhancers, and broad H3K4me3 domains.

**Electronic supplementary material:**

The online version of this article (10.1186/s13059-018-1430-4) contains supplementary material, which is available to authorized users.

## Background

The local chromatin conformation regulates gene transcriptional activity through facilitating interactions between promoters and distant active regulatory elements such as enhancers, repressors, and silencers [1, 2]. These *cis*-regulatory elements are loosely packed and relatively free of nucleosomes, which are necessary for transcription factors and other regulatory proteins to gain access to DNA [3–5]. Traditionally, active regulatory elements (open chromatin) can be assayed genome-wide by DNase-hypersensitive sites identified by sequencing (DNase-seq) or formaldehyde-assisted isolation of regulatory elements by sequencing (FAIRE-seq) [6, 7].

Recently, several elaborate methods to identify chromatin interaction maps have been developed, including chromatin interaction analysis by paired-end tag sequencing (ChIA-PET) [8] and chromosome conformation capture (3C)-based methods [9], such as 4C [10, 11], 5C [12], Hi-C [13], in situ Hi-C [14], Capture-C [15], DNase-C [16], Micro-C [17], single-cell Hi-C [18, 19], HiChIP [20], and PLAC-seq [21]. In particular, DNase-C identifies high-confidence DNA contacts at kilobase resolution by using DNase I to digest the genome DNA instead of restriction enzymes [16, 22]. These techniques have greatly advanced our understanding of detailed features of the genome 3D structure and regulation of the genome [6, 23–26]. However, ChIA-PET, HiChIP, and PLAC-seq only determine the subset of interactions mediated by specific DNA-binding proteins, whereas Hi-C captures all genomic interactions indiscriminately, which may flood important contacts between open chromatin and distal regulatory elements.

In order to overcome these limitations, we integrated the FAIRE-seq and in situ Hi-C assays and developed the open chromatin enrichment and network Hi-C (OCEAN-C) method for mapping global open chromatin interactions. By aggregating open chromatin associated with interacting partners through direct phenol-chloroform extraction, OCEAN-C enriched interactions among active *cis*-regulatory elements, which mainly occurred among promoters and enhancers and thus regulated gene transcription. OCEAN-C is a novel tool for studying open chromatin interactions and their relationship with gene regulation.

## Results

### Genome-wide open chromatin interaction assay using OCEAN-C

**Fig. 1:**
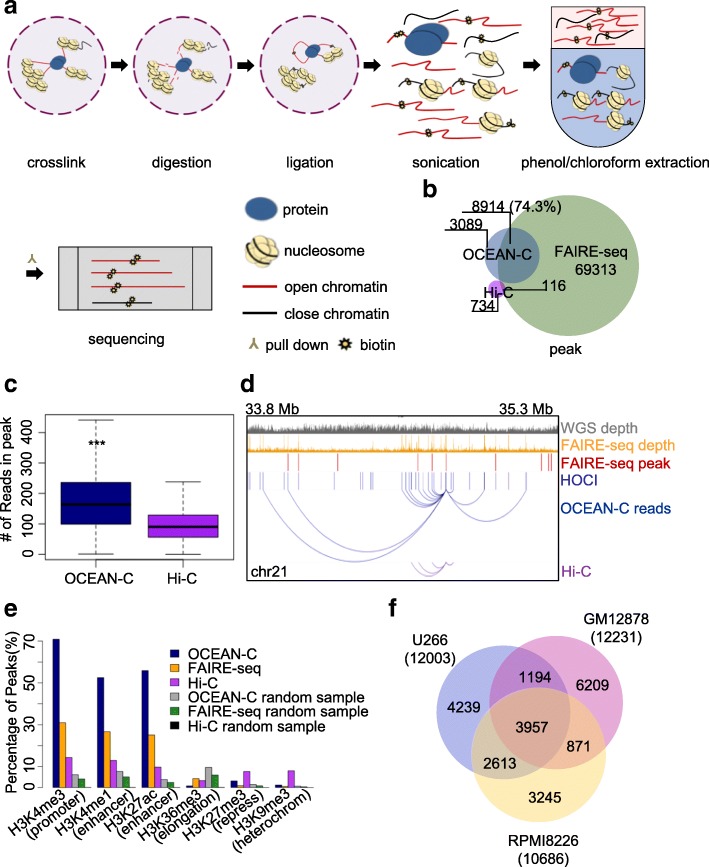
Open chromatin enrichment and network Hi-C (OCEAN-C) identifies hubs of open chromatin interactions (HOCIs) without the need for antibodies. **a** The OCEAN-C method. **b** Venn diagram of peaks determined by FAIRE-seq, OCEAN-C, and Hi-C methods in U266 cells using the ZINBA algorithm. **c** Boxplots showing the distributions of OCEAN-C and Hi-C reads in OCEAN-C peaks of U266 cells. **d** An example of chromatin interactions involving HOCIs. The browser view of a 1.5-Mb region shows a randomly chosen HOCI with associated OCEAN-C and Hi-C interactions. Each loop indicates a unique valid read pair. **e** Percentage of peaks or random regions of OCEAN-C, Hi-C, and FAIRE-seq that overlap with various histone modification markers (U266). **f** Venn diagram of HOCIs called from three cell lines (U266, RPMI-8226, and GM12878)

We first performed in situ Hi-C and FAIRE-seq experiments using U266 multiple myeloma cells to identify genome-wide chromatin interactions and open chromatin regions [14, 27]. As expected, our data exhibited high reproducibility and typical features of Hi-C and FAIRE-seq results (Additional file 1: Figure S1, Additional file 2: Table S1). Next, we developed the OCEAN-C assay by integrating the in situ Hi-C and FAIRE-seq protocols. A step for the phenol-chloroform extraction of nucleosome-depleted chromatin (open chromatin) was added after the biotinylated residue addition and sonication steps of Hi-C, enabling the specific enrichment of nucleosome-free DNAs and DNA fragments that re-ligated with the open chromatin (Fig. 1a, “Methods”). The ratio of isolated OCEAN-C DNA with respect to total genomic DNA is 1–3%, which is similar to FAIRE-seq [27]. The biotin-labeled DNA fragments were then enriched from the extracted OCEAN-C DNA and followed by library construction and high-throughput sequencing. Open chromatin regions that form peaks due to multiple chromatin interactions were then called by the ZINBA algorithm [28] used for FAIRE-seq peak identification.

We identified 12,003 OCEAN-C peaks (median of broad size was 1.4 kb and of narrow size 232 bp) with 43.4 million valid read pairs standing for intra-chromsomal interactions in the U266 cell line. Of these, 74.3% overlapped with FAIRE-seq peaks; in contrast, only 850 peaks were determined from the same number of Hi-C reads, which barely had any intersection with OCEAN-C or FAIRE-seq peaks (Fig. 1b). The high ratio of overlap with FAIRE-seq peaks confirmed that the peak regions determined by OCEAN-C are open chromatin regions. Moreover, the OCEAN-C peaks only comprise a small portion (approximately 13%) of the total number of open chromatin regions identified by FAIRE-seq, indicating that most open chromatin regions do not show a significantly higher interaction frequency than other regions. We observed 174 interactions per OCEAN-C peak on average (Fig. 1c), which is significantly higher than the number for Hi-C data (*p* value < 2.2e-16). Therefore, OCEAN-C peaks represent chromatin interaction hubs that form multiple interactions with a set of loci along the chromosome (Fig. 1d and Additional file 1: Figure S2A), and we name these regions hubs of open chromatin interactions (HOCIs). Correlation analysis using epigenetic markers revealed that HOCIs are mainly occupied by active histone modifications (H3K4me3, approximately 70%; H3K4me1, approximately 50%; and H3K27ac, approximately 50%) at percentages that remarkably exceed those of open chromatin identified by FAIRE-seq and Hi-C peaks (Fig. 1e), demonstrating that HOCIs are mainly active *cis*-acting elements, especially promoters (H3K4me3) and enhancers (H3K4me1 and H3K27ac).

To further test the reproducibility and feasibility of OCEAN-C, we examined the method in RPMI-8226 multiple myeloma cells and GM12878 lymphoblastoid cells. The three cell lines exhibited similar numbers of HOCIs and similar histone modification properties, demonstrating that HOCIs represent a common phenomenon in different cell lines (Fig. 1f and Additional file 1: Figure S2B). The large difference in the locations of HOCIs between different cell lines is suggestive of specific open chromatin interactions that are associated with gene regulation. Next, we compared the results of OCEAN-C and in situ Hi-C in identifying large-scale chromatin architectures such as topologically associated domains (TADs) and compartments and found that interaction heat maps, TADs, and A/B compartments exhibited high concordance between OCEAN-C and Hi-C (Additional file 1: Figure S2C–F), demonstrating the ability of OCEAN-C to identify the same TADs and A/B compartments as in situ Hi-C. Furthermore, we evaluated the effect of sequencing depth and software packages used on peak calling. The number of HOCIs identified was affected by low sequencing depth and gradually became saturated with increasing read number (Additional file 1: Figure S3A). By using the MACS2 software to call peaks from the OCEAN-C data of U266 cells, we obtained 9926 peaks, 4718 of which overlapped ZINBA-identified peaks, suggesting that the peak signals of open chromatin in OCEAN-C data can be detected by different algorithms and combining different peak-calling methods may be helpful to identify reliable HOCIs (Additional file 1: Figure S3B–E).

We also compared OCEAN-C with the DNase-C technique in identifying open chromatin interactions (Additional file 1: Figure S4). The results showed that while DNase-C method captures open-chromatin interactions at fine-scale, OCEAN-C performs better than DNase-C in peak calling and identifying accurate open chromatin interaction peaks.

### HOCIs are bound by a cluster of DNA-binding proteins

**Fig. 2:**
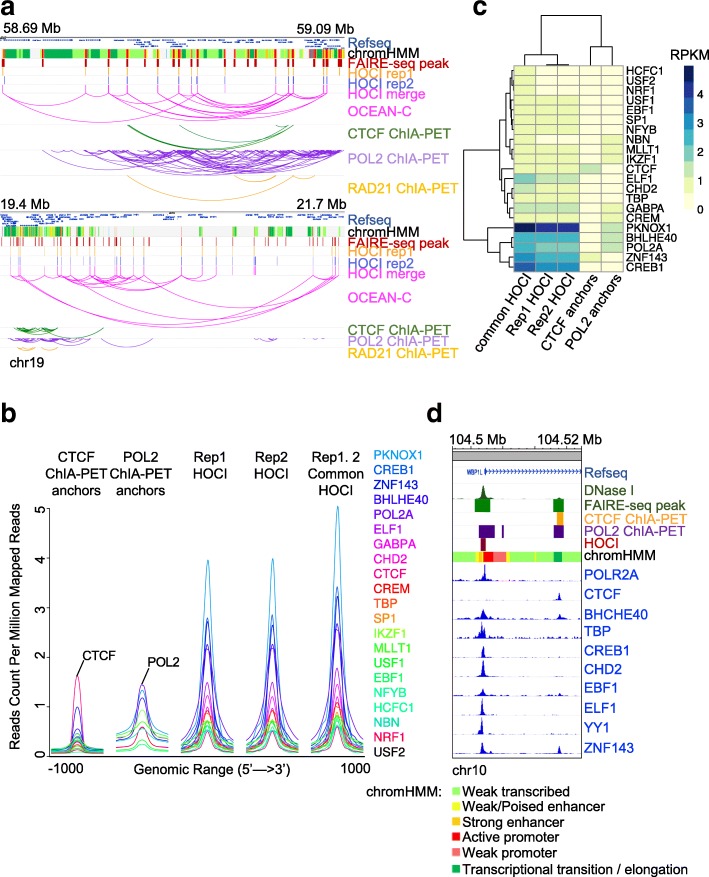
HOCIs are bound by a cluster of DNA-binding proteins. **a** Comparison between OCEAN-C and ChIA-PET data (GM12878). The browser view of two genomic regions shows interactions among HOCIs and ChIA-PET anchors. **b** Density plot of ChIP-seq data for 21 DNA-binding proteins at ChIA-PET anchors or HOCIs (GM12878). **c** Hierarchical clustering of binding signals for 21 DNA-binding proteins at ChIA-PET anchors or HOCIs (GM12878). **d** An example region containing a HOCI and ChIA-PET anchors is shown with DNase I read depth and ten ChIP-seq signals (GM12878)

Previous studies revealed that chromatins form loops at approximate kilobase-scale resolution with the binding of scaffold proteins such as CTCF and cohesin, which facilitate gene regulation [14, 26]. These studies were primarily based on saturated sequencing of Hi-C data or protein-based chromatin interaction analyses such as ChIA-PET, HiChIP, or PLAC-seq. We compared the HOCIs identified by OCEAN-C with anchors determined by ChIA-PET and loops determined by Hi-C in GM12878 cells. Both intersecting and distinct HOCIs were identified compared with ChIA-PET results (Fig. 2a). About 41% HOCIs overlapped with CTCF loop anchors determined by CTCF ChIA-PET, and 47% HOCIs overlapped with anchors determined by Pol II ChIA-PET; in contrast, only 21% of the HOCIs were loop regions determined by Hi-C (Additional file 3: Table S2A). The overlap proportions demonstrate the ability of OCEAN-C in identifying kilobase-scale loop anchors. More importantly, the non-overlapping proportion demonstrates the specificity of the OCEAN-C method. While a pair of anchors from ChIA-PET mainly interact with each other, a HOCI interacts with a set of loci, including loop interactions (Figs. 1d and 2a). To further confirm the interactions between HOCIs, we selected two clusters of HOCIs and performed 3C validation experiment. The results showed that over half of pairwise interactions among HOCIs of both clusters are detected by the 3C method (Additional file 1: Figure S5), demonstrating the reliability of HOCI interactions discovered by OCEAN-C.

As OCEAN-C is designed to capture interactions between open chromatin regions without relying on specific antibodies, we speculated that HOCIs are chromatin regions bound by multiple DNA-binding proteins. To confirm this hypothesis, we integrated ChIP-seq data from ENCODE, ChIA-PET, and OCEAN-C data of GM12878 cells. As expected, chromatin anchors identified by CTCF ChIA-PET displayed much stronger CTCF ChIP-seq signals than any other DNA-binding proteins, and Pol II also exhibited the strongest binding signal at anchors of Pol II ChIA-PET (Fig. 2b), demonstrating the enrichment of specific protein-binding regions in ChIA-PET experiments. In contrast, HOCIs displayed enriched binding signals for a larger set of DNA-binding proteins, including active transcription factors (PKNOX1, Pol II), transcription repressors (BHLHE40, SP1, YY1), transcription regulators (ZNF143, CREB1, GABPA), and CTCF (Fig. 2b). Moreover, several lymphoid cell-specific transcription factors showed strong binding signals, including E74-like factor 1 (ELF1) and Early B-cell factor 1 (EBF1), demonstrating the ability of OCEAN-C to identify key lineage-specific DNA-binding proteins (Fig. 2b). Specifically, the B-cell-specific transcription factor ELF1 showed higher binding signal at HOCIs than other factors except Pol II-related proteins (POL2A, PKNOX1, BHLHE40, ZNF143, and CREB1; Fig. 2c).

On average, a HOCI is occupied by 9.1 different DNA-binding proteins, compared to an average of 6.7, 5.3, and 6.5 different DNA-binding proteins occupying a Pol II ChIA-PET anchor, CTCF ChIA-PET anchor, and Hi-C loop anchor, respectively (Additional file 1: Figure S6). Moreover, the ChIA-PET and Hi-C loop anchors overlapping HOCIs were bound by significantly more DNA-binding proteins than the other anchors (*t*-test, *p* value < 2.2e-16; Additional file 1: Figure S6B), demonstrating that ChIA-PET can only capture a portion of HOCIs, which were DNA loop anchors occupied by both ChIA-PET anchor proteins and other DNA-binding proteins. In addition, contour plots showed that HOCIs had shorter width and more binding proteins overall, while most POL2/CTCF ChIA-PET anchors were longer and occupied by less than five different DNA-binding proteins (Additional file 1: Figure S6C). We also analyzed the DNA sequence motifs of HOCIs and ChIA-PET anchors. CTCF ChIA-PET anchors showed extremely enriched CTCF/CTCFL DNA binding motifs, while HOCIs showed less difference in the significance level of the top five enriched motifs, including CTCF/CTCFL (Additional file 1: Figure S6D). Specifically, at the locus of the gene *WBP1L*, two regions were identified as open chromatin regions by FAIRE-seq, one near the promoter and the other in close proximity to the promoter within the gene body (Fig. 2d). The promoter of *WBP1L* was identified as a HOCI by OCEAN-C and confirmed by strong binding signals for many DNA-binding proteins, including Pol II but not CTCF, while the second open chromatin region was not identified as a HOCI due to the binding signals of mainly CTCF and Pol II but not other proteins (Fig. 2d). Therefore, the occupancy of multiple proteins and frequent interactions with other chromatin regions distinguishes HOCIs from other open chromatin regions.

To further explore the genomic properties of HOCIs, we analyzed the chromatin states of HOCIs as well as anchors of CTCF or Pol II ChIA-PET in GM12878 cells (Additional file 1: Figure S7A). CTCF anchors were mainly marked as insulators, and Pol II anchors were mainly marked as promoters and enhancers, consistent with the biological function of these two proteins. HOCIs were most commonly identified as promoters (approximately 50%), followed by enhancers (approximately 15%), and insulators (approximately 15%). We clustered HOCIs according to their binding signals of multiple DNA-binding proteins. The results showed that promoter and enhancer HOCIs are occupied by many proteins, whereas insulator HOCIs are occupied by a few proteins, including CTCF, ZNF143, EBF1, and BHLHE40 (Additional file 1: Figure S7B). Meanwhile, HOCIs located within inactive chromatin regions had few interactions with DNA-binding proteins (Additional file 1: Figure S7B). Taken together, these results indicate that HOCIs identified by OCEAN-C are mainly functional *cis*-regulatory elements that are bound by a cluster of regulatory proteins.

### HOCIs form promoter- and enhancer-based topological architectures that associate with gene expression

**Fig. 3:**
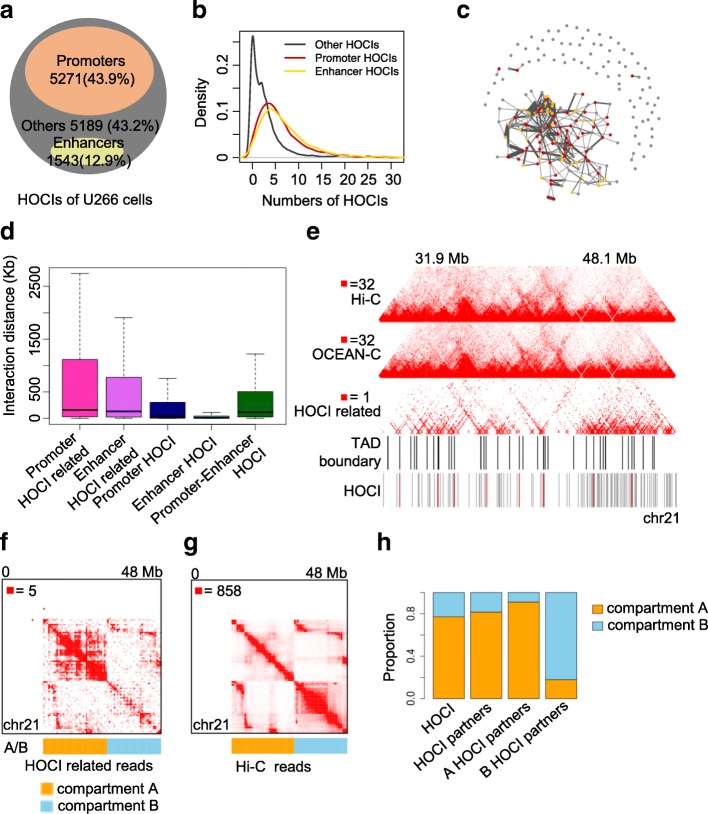
Characteristics of HOCIs. **a** The proportion of three types of HOCIs (U266): promoters, enhancers, and others. **b** Density distribution of the number of HOCIs interacting with different types of HOCIs. **c** Interaction network formed by HOCIs in chromosome 21 (U266). *Red node*, promoter HOCI; *yellow*, enhancer HOCI; *gray*, other HOCI. The thickness of *edges* indicates the interaction intensity between two HOCIs. **d** Interaction distance between HOCIs and their interacting regions. ‘Promoter HOCI related’ means that at least one end of a valid read pair is mapped to promoter HOCIs; ‘enhancer HOCI related’ means that at least one end of a read pair is mapped to enhancer HOCIs; when both ends of a read pair belong to promoter HOCIs or enhancer HOCIs, the read pair is classified as ‘Promoter HOCI’ and ‘enhancer HOCI’, respectively; when two ends of a read pair separately map to a promoter HOCI and an enhancer HOCI, the read pair is classified as ‘Promoter-Enhancer HOCI’. **e** Heat maps showing Hi-C, OCEAN-C, and HOCI-related reads in chromosome 21 at 40 kb resolution. **f** Heat map of HOCI-related reads in chromosome 21 (U266) at 40-kb resolution with bins reordered by A/B compartments. Only valid pairs with at least one end mapped to HOCIs are defined as HOCI-related reads and used to generate the interaction heat map. **g** Heat map of Hi-C reads in chromosome 21 (U266) at 40-kb resolution with bins reordered by A/B compartments. **h** Proportions of HOCIs located in compartment A/B (U266). *HOCI* stands for all the HOCIs detected by OCEAN-C, *HOCI partners* indicate other loci that interact with all HOCIs or those HOCIs located in A or B compartments

To further investigate the biological functions of HOCIs, we explored the chromatin interactions involved with HOCIs and their relationship with gene transcription. Similar to GM12878 cells (Additional file 1: Figure S7A), the majority of HOCIs in U266 cells were promoters (44%) and enhancers (13%), as classified according to histone modifications (Fig. 3a). Most HOCIs also interacted with other HOCIs (six on average; Fig. 3b) and therefore formed an interaction network including promoters, enhancers, and other *cis*-regulatory elements across the entire chromosome (Fig. 3c and Additional file 1: Figure S8). We calculated the chromosomal distances spanned by these interactions, and most interactions related to promoter HOCIs and enhancer HOCIs occurred within 500 kb, with a few interactions spanning several megabases (Fig. 3d), consistent with the findings of a previous study using Capture-C [15]. Interactions within promoter HOCIs or enhancer HOCIs covered significantly shorter chromosomal distances, with median distances of 44 and 13 kb, respectively, whereas interactions between promoter HOCIs and enhancer HOCIs had a longer median span of 117 kb (Fig. 3d).

We next explored the location of HOCIs relative to the hierarchical spatial structures of the genome, including topological associated domains (TADs) and A/B compartments. HOCIs preferentially occurred at TAD boundaries (Fig. 3e, Additional file 3: Table S2B), and HOCI-mediated interactions were mainly within active A compartments (Fig. 3f, h); in contrast, Hi-C interactions occurred abundantly within both A and B compartments (Fig. 3g). These results suggest that HOCI-mediated interactions preferentially involve active chromatin regions, especially TAD boundaries.

**Fig. 4:**
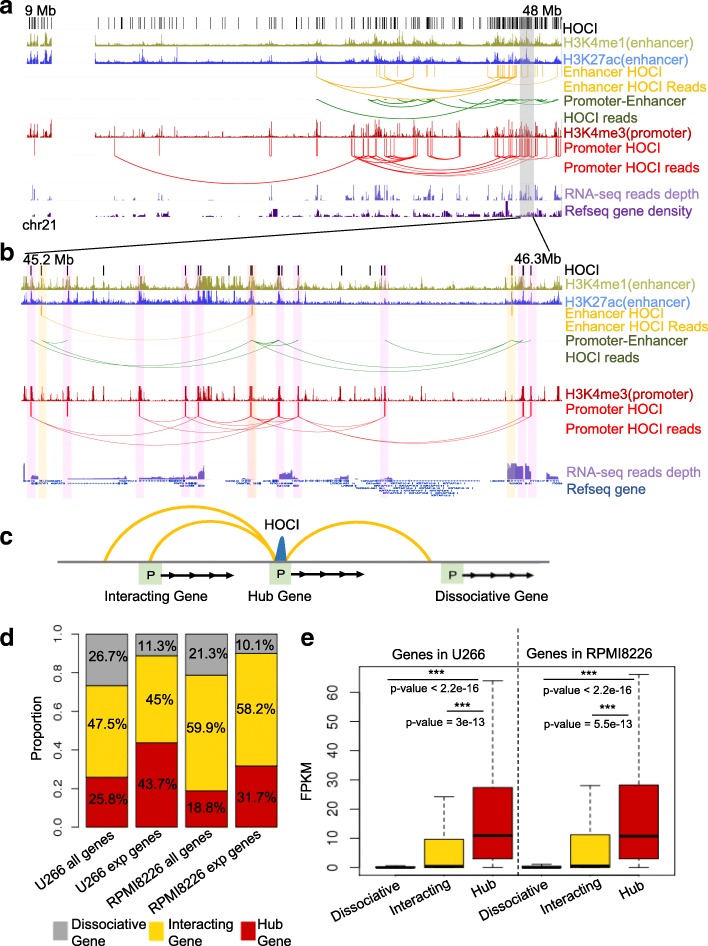
The association between HOCIs and gene expression. **a** The browser view of a 40-Mb region showing the relationship between HOCI interactions and gene transcription levels. *Loops* indicate read pairs (GM12878 cell). **b** Magnification of the region highlighted in *gray* in **a**. **c** The model of three different types of genes. *Hub gene*, the promoter is a HOCI; *interacting gene*, the promoter interacts with a HOCI; *dissociative gene*, the promoter has no interaction with a HOCI. **d** The proportion of the three gene types within all genes or transcribed genes (“exp genes”). **e** Comparison of the transcription levels of the three types of genes in U266 and RPMI-8226 cells

To further explore the relationship between HOCI interactions and gene transcription, we randomly selected a chromatin region (chromosome 21, 9–48 Mb) and plotted the chromatin interactions involving HOCIs and the read depth of RNA-seq experiments in U266 cells (Fig. 4a, b). Genes forming promoter–enhancer interactions through HOCI interaction networks were highly transcribed; in contrast, genes without HOCI-mediated interactions were hardly transcribed. Gene-rich regions form more intensive HOCI interactions than gene-poor regions (Fig. 4a, b). We next categorized genes into three groups according to their local open chromatin interactions as follows (Fig. 4c): genes whose promoters were HOCIs (hub genes), genes whose promoters were not HOCIs but interacted with HOCIs (interacting genes), and genes whose promoters were not involved in HOCI interactions (dissociative genes). These three types of genes exhibited significant differences at the transcription level (Fig. 4d, e and Additional file 3: Table S2C, D). Most expressed genes (~ 90%) were either hub genes or interacting genes. The hub genes were expressed at a significantly higher expression level than genes of the two other groups, and dissociative genes showed the lowest expression level (Fig. 4e). Furthermore, housekeeping genes comprised a higher proportion of hub genes than the expressed genes (Additional file 3: Table S2D). These results demonstrate the key roles of HOCIs in forming promoter and enhancer chromatin interactions that are crucial for gene transcription.

### HOCI-mediated interactions explain differential gene expression

**Fig. 5:**
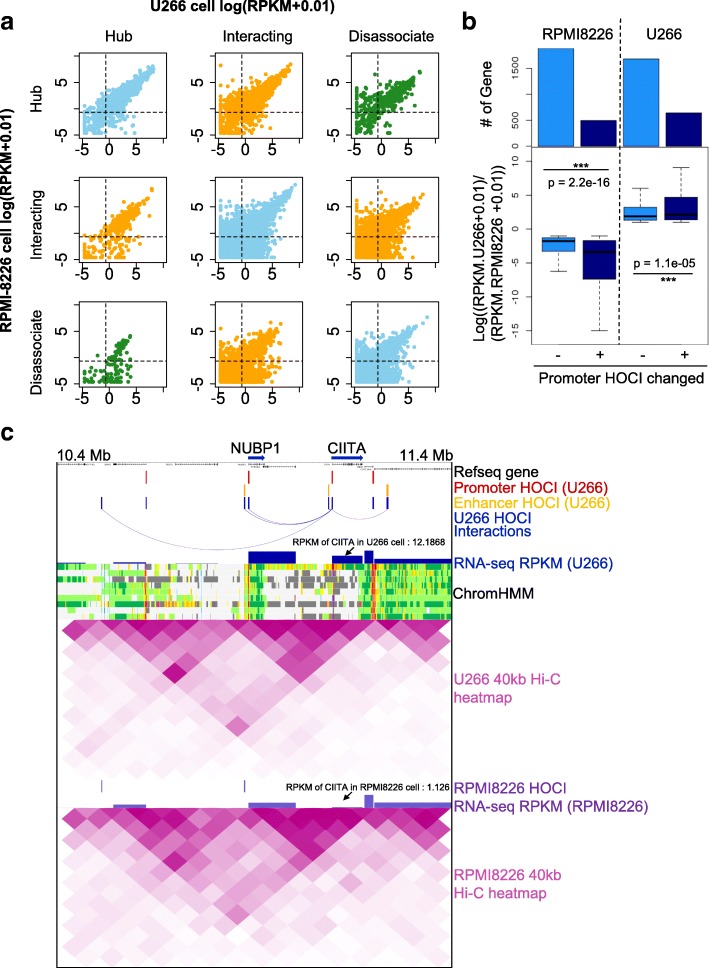
HOCI interactions explain differential gene expression. **a** Scatter plot showing the RNA-seq expression of different types of genes in U266 vs RPMI-8226 cells. *Blue*, genes belonging to the same type in the two cell lines; *green*, genes whose type is hub in one cell line and dissociative in the other; *orange*, genes of other type changes; *dashed lines*, cutoff for expressed genes at RPKM > 0.5. **b** *Top*: barplots showing the number of genes in each category. “*Promoter HOCI changed*” are the genes whose promoters overlap with HOCIs in one cell line but do not overlap with HOCIs in the other cell line. *Bottom*: boxplots showing the change of transcription levels in the down-regulated (*left*) or up-regulated (*right*) gene groups. **c** The browser view of a 1-Mb genomic region showing the *CIITA* gene’s promoter has HOCI interactions in U266 cells but not in RPMI-8226 cells, which explains the gene’s differential transcription between these two cell lines

We further investigated whether changes in HOCIs can explain differential gene transcription between different cell lines. We compared the gene transcription levels of two multiple myeloma cell lines (U266 and RPMI-8226) according to the three gene types defined above. Genes that have different types between the two cell lines showed significantly different gene expression, while genes that have the same types between the two cell lines showed similar transcription levels (Fig. 5a). Large decreases in transcription occurred with the disruption of HOCIs, whereas significant increases in transcription occurred with the formation of HOCIs (Additional file 1: Figure S9). In particular, a gene tended to lose transcription completely when it transformed from a hub type to a dissociative type. This was further confirmed via comparisons between differentially expressed genes that can or cannot be explained by the change of HOCI-mediated interactions at promoters (Fig. 5b). Genes with differential HOCI-mediated interactions showed significantly greater differential expression than those with no interaction changes.

To specifically illustrate the relationship between open chromatin interactions and gene expression, we selected one differentially expressed gene, Class II major histocompatibility complex transactivator (*CIITA*), an important gene that participates in B-cell differentiation, and examined the nearby open chromatin interactions, Hi-C heat maps, and RNA-expression levels (Fig. 5c). In U266 cells, the promoter of *CIITA* was identified as a HOCI that forms multiple interactions with nearby genes, associating with high expression of the gene, whereas such HOCIs and interactions were not detected in RPMI8226 cells, associating with a weak transcription signal of the gene. In contrast, Hi-C heat maps cannot detect such differences at 40-kb resolution. Taken together, we demonstrated that OCEAN-C identified HOCI-mediated open chromatin interactions that are crucial for gene transcription and changes.

### Most super-enhancers and many broad H3K4me3 domains overlap with HOCIs

**Fig. 6:**
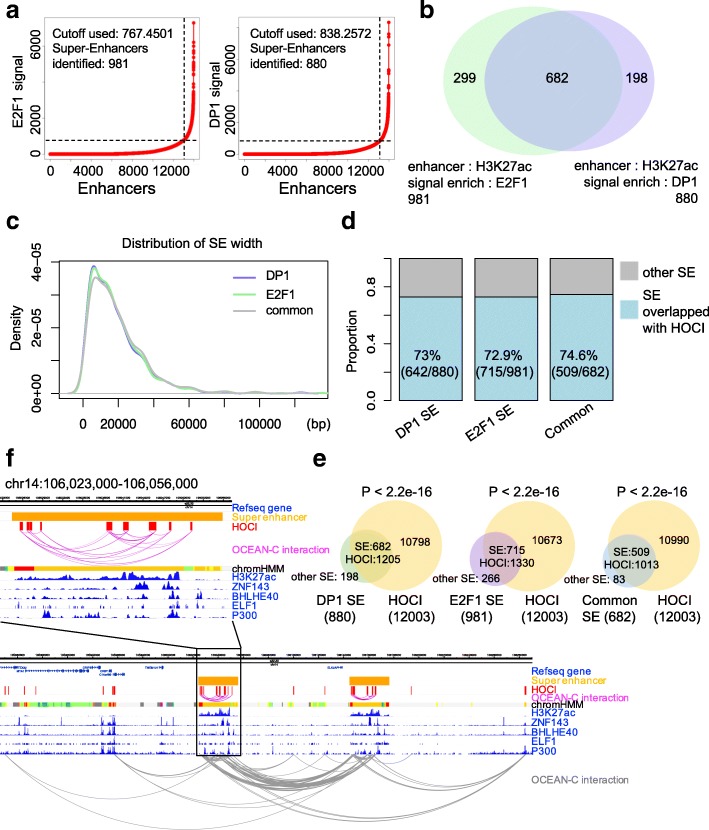
Overlaps between HOCIs and super-enhancers. **a** Identification of super-enhancers in U266 cells based on E2F1 or DP1 signals. **b** Venn diagram showing the overlap between the two types of super-enhancers defined by E2F1 or DP1 signals (U266). **c** The length distribution of the two types of super-enhancers defined by E2F1 or DP1 signals (U266). *SE* super-enhancer. **d** Proportions of super-enhancers overlapping HOCIs. **e** Venn diagram showing the overlap between super-enhancers and HOCIs. The *p* values were generated using Fisher test. **f** The browser view of a genomic region in chromosome 14, showing HOCI-mediated chromatin interactions within and between super-enhancers (GM12878). OCEAN-C interaction: both ends of OCEAN-C read pairs mapped to HOCIs. *Pink*, interactions between super-enhancer-related enhancer HOCIs; *gray*, all interactions

Super-enhancers are defined by exceptional enrichment of master transcription factor binding or active chromatin markers determined by ChIP-seq, and they confer high transcriptional activity to nearby genes [29, 30]. Since super-enhancers are relatively broad open chromatin regions that participate in gene regulation through chromatin interaction [29, 30] and OCEAN-C captures open-chromatin interactions, we speculated that HOCIs overlap with super-enhancers. The interaction distances among enhancer HOCIs are significantly shorter than other types of HOCI interactions, indicating that enhancer HOCIs may form super-enhancers (Fig. 3d). To confirm this hypothesis, we defined super-enhancers in U266 cells through ChIP-seq data of H3K27Ac, E2F1, and DP1 following previous instructions (Fig. 6a-c). Among the 880 super-enhancers defined by H3K27ac/DP1, 642 (73%) overlapped with HOCIs; among the 981 super-enhancers defined by H3K27ac/E2F1, 715 (72.9%) overlapped with HOCIs, demonstrating that most super-enhancers are composed of HOCIs (Fig. 6d, e). Interestingly, super-enhancers formed interactions with themselves and with different super-enhancers through the interactions of HOCIs (Fig. 6f). These results demonstrate that most super-enhancers are composed of HOCIs and OCEAN-C is capable of identifying super-enhancers and their interactions.

**Fig. 7:**
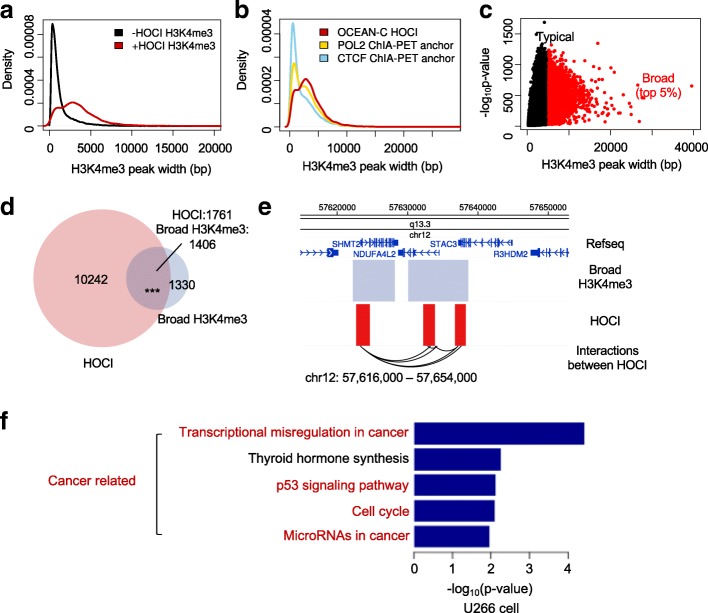
Overlaps between HOCIs and broad H3K4me3 domains. **a** The width distribution of H3K4me3 peaks. *Red*, H3K4me3 peaks overlapped with HOCIs; *black*, not overlapped with HOCIs (GM12878). **b** The width distribution of H3K4me3 peaks. *Red*, overlapping HOCIs; *gold*, overlapping POL2 ChIA-PET anchors; *blue*, overlapping CTCF ChIA-PET anchors (GM12878). **c** The -log_10_*p*-value of H3K4me3 peaks (*y-axis*) are plotted against peak width (*x-axis*). *Black* and *red dots* indicate typical and broad H3K4me3 peaks, respectively (U266). **d** Venn diagram of HOCIs and broad H3K4me3 peaks (U266). **e** Browser view of a genomic region in chromosome 12, showing the interactions within and between broad H3K4me3 regions (U266). *Interactions between HOCI*, both ends of OCEAN-C read pairs mapped to HOCIs. **f** KEGG pathway enrichment analysis of genes whose promoters are associated with both HOCIs and broad H3K4me3 peaks, while using all genes whose promoters are associated with HOCIs as background

Broad H3K4me3 domains (wider than 4 kb) are associated with increased transcription elongation and enhancer activities, especially at tumor suppressor genes, and form chromatin interactions with super-enhancers [31, 32]. In GM12878 cells, H3K4me3 regions overlapping with HOCIs showed broader signals compared with the rest of the H3K4me3 regions or the H3K4me3 regions overlapping with ChIA-PET anchors (Fig. 7a, b), suggesting the enrichment of long H3K4me3 peaks in HOCIs. We next analyzed the relationship between HOCIs and broad H3K4me3 domains, which are potentially long open chromatin regions. We defined 2736 broad H3K4me3 regions in U266 cells and 51.4% (1406) of them overlapped with HOCIs (Fig. 7c, d). Most broad H3K4me3 regions contained one to five interacting HOCIs. Specifically, two nearby broad H3K4me3 regions at chr12:57620000–57,640,000 interacted with each other through the three HOCIs within them (Fig. 7e). In addition, we performed pathway enrichment analysis of the genes whose promoters overlap with both HOCIs and broad H3K4me3 domains, and found that four out of the five top enriched pathways were related to cancer (Fig. 7f). These results demonstrate that many broad H3K4me3 domains are composed of HOCIs and OCEAN-C is capable of identifying broad H3K4me3 domains and their interactions.

## Discussion

Hi-C-based methods and ChIA-PET have greatly advanced our understanding of the 3D architecture of the nucleus by uncovering TADs, compartments, and chromatin loops. Previous ChIA-PET studies illustrate that promoter–promoter interactions provide a topological basis for transcriptional regulation, and CTCF and cohesin mediate the formation of 3D genome architectures [26, 33]. Several algorithms have been developed to discover chromatin interaction structures such as chromatin interaction hubs [34], long-range interaction networks [35], inter-chromosomal chromatin clusters [36], and active promoter–enhancer associations [37] by integrating Hi-C data with epigenome and transcriptome data. However, Hi-C requires billions of reads to detect loops, while ChIA-PET and HiChIP are antibody-dependent and thus only capture DNA interactions mediated by specific proteins. Capture-C mainly captures interactions directly involving promoters. To overcome these limitations, we developed the OCEAN-C method, which enriches open chromatin interactions through phenol-chloroform extraction without using antibodies. OCEAN-C can identify sharp open chromatin regions interacting with many other chromatin regions, which we define as HOCIs, and facilitates the study of open chromatin interactions. We show that OCEAN-C is reproducible, time saving (~ 3 days), and has low sequencing costs (~ 100 million read pairs are sufficient to identify 10,000 HOCIs along with TADs and compartments).

The conformation of *cis*-regulatory elements is as important as their primary sequences with regard to gene regulation. It is important to explore the interactions among *cis*-regulatory elements such as enhancers, promoters, and insulators to understand how they regulate gene expression. Based on OCEAN-C data, we identify HOCIs as open chromatin interaction hubs with potential regulatory functions. We demonstrate that HOCIs preferentially form open chromatin interactions, including promoter–enhancer, promoter–promoter, and enhancer–enhancer interactions, which distinguish HOCIs from other open chromatin regions. A HOCI often mediates clustered chromatin interactions and can be important for coordinated transcription of multiple genes that are nearby and faraway. In addition, OCEAN-C is feasible for investigating changes in open chromatin conformations, such as promoter–enhancer interactions, that result in differential gene expression. We demonstrate that hub genes whose promoters are HOCIs display the highest transcription activity, and changes in HOCIs between different cell lines are associated with marked changes of transcription. These findings suggest that OCEAN-C is a suitable tool for studying the activation or inactivation of developmental genes or cancer genes due to the changes in chromatin conformation.

Despite these advantages, the current version of OCEAN-C has several areas that could be improved. First, OCEAN-C is based on the Hi-C method, which only captures chromatin interactions near recognition sites of the specific restriction enzyme used. Although the four-base restriction enzymes we used have abundant cutting sites along the genome, they may miss capturing certain chromatin regions. Second, because 1–3% of the total DNA was extracted as chromatin interactions related to open chromatin, OCEAN-C needs ~ 1 million cells in order to obtain sufficient DNA for library construction, which restricts its application for clinical samples. We will continue to develop and improve OCEAN-C to overcome these limitations.

## Conclusions

We demonstrate that OCEAN-C is a powerful method for investigating open chromatin interactions and the dynamic of HOCIs in regulating gene transcription.

## Methods

### Cell culture and collection

U266 cells (ATCC TIB-196), RPMI8226 cells (ATCC CCL-155), and GM12878 cells were grown in RPMI-1640 medium containing 10% fetal bovine serum at 37°C and 5% CO_2_. The cells were cultured to 80–90% confluence and then collected and washed once with PBS. For crosslinking cells, formaldehyde was added at a final concentration of 1% at room temperature (RT) for 10 min, and then quenched with glycine (0.2 M) for 5 min. The crosslinked cells were washed once with PBS, flash-frozen by liquid nitrogen, and stored at − 80 °C for further usage.

### FAIRE-seq and in situ Hi-C experiments

These two experiments were performed strictly in line with previously reported protocols [14, 27]. For FAIRE-seq data, reads were mapped to the hg19 assembly by bwa-mem, filtering was performed by removing unmapped and duplicated reads, and open chromatin peaks were determined by ZINBA [28] using the following parameters: input = none, offset = 50, method = “mixture,” peak confidence = 0.95, numProc = 4, buildwin = 1, refinepeaks = 1, selected model = T, tol = 1 × 10 − 5, and others as default. The “--broad” parameter was set to TRUE when calling broad peaks by the “callpeak” function of ZINBA. For Hi-C data, reads were trimmed to 36 bp and aligned to the hg19 assembly by bowtie2. Only uniquely mapped read pairs (MAPQ > 1) were kept, filtering was performed following previous protocols, the interaction matrix was normalized by the ICE method, and TADs and A/B compartments were identified using the HiTC package.

### RNA-seq experiment

U266 and RPMI8226 cells were cultured to 80–90% confluence and harvested. RNA purification and library construction were performed by Novogene (Beijing, China) with three independent replicates for each cell line, and the differential expression analysis was performed using the TopHat-cufflinks software with the recommended parameters.

### OCEAN-C experiment

#### Cell fixation, digestion, and re-ligation

Digestion with the MboI enzyme, filling-in with biotin-labeled dATP, and re-ligation by the T4 ligase were performed using fixed cells (2–5 × 10^6^ cells) following the instructions of the in situ Hi-C method.

#### Cell sonication

Cells were resuspended in 2 ml lysis buffer (10 mM Tris-HCl [pH 8.0], 2% Triton X-100, 1% SDS, 100 mM NaCl, and 1 mM EDTA) and sonicated to an average DNA fragment size of 300–400 bp (Branson Sonifier 450D). The results for each 30 s of sonication were checked under a microscope until no intact cells were observed. The cells were kept on ice and foaming was avoided. The efficiency of sonication was further confirmed by agarose gels of purified DNA from a portion (5%, 100 μl) of the cell lysate.

#### Open chromatin purification

The supernatants were transfered to new 1.5 ml tubes after centrifugation (15,000-20,000×g for 5 min at 4°C). To purify the open chromatin, 1 volume phenol-chloroform-isoamyl alcohol was added to each aliquot of cell lysate. After vortexing for 10 s, each aliquot was centrifuged at 13,000×g for 5 min, and the top layer was transferred to a fresh 1.5 ml tube. The phenol–chloroform–isoamyl alcohol extraction step was repeated once, after which 200 μl of chloroform–isoamyl alcohol were added to each tube to remove traces of phenol, and the aqueous layer was transferred to a new 1.5-ml tube. Next, a 1/10 volume of 3 M sodium acetate (pH 5.2), 2 volumes of 95% ethanol, and 1 μl of 20 mg/ml glycogen were added to each tube and incubated at − 80 °C for 30 min (or longer) after fully mixing the sample. Each pellet was centrifuged at 13,000×g for 15 min at 4 °C, and the DNA pellet was washed twice with 500 μl of ice-cold 70% ethanol. The DNA was dried by leaving tubes open for 5 min and re-suspended in 200 μl of 10 mM Tris-HCl (pH 7.4).

#### Reverse cross-linking and DNA quantification

DNase-free RNase A (1 μl) was added following 30 min of incubation at 37 °C, and 1 μl of proteinase K was added and incubated at 55 °C for 1 h and then at 65 °C overnight to reverse cross-linking. The DNA was collected by adding 0.9 volume of AMPure XP beads (Beckman Coulter, A63881) and washed with 300 μl 10 mM Tris-HCl (pH 7.4). The concentration of DNA was measured by Qubit. The amount of purified DNA should not exceed 5% of total genomic DNA (1–3%). An optimized step can be performed to boost the yield before the biotin pull-down operation by sonicating the purified DNA with Covaris to a median fragment size of 300–500 bp.

#### Biotin pull-down

Myone Streptavidin T1 beads (150 μl; Life technologies) were washed once with 400 μl 1 × TWB (5 mM Tris-HCl (pH 7.5), 0.05 mM EDTA, 1 M NaCl, 0.05% Tween 20), separated on a magnet, and resuspended with 300 μl 2× binding buffer (10 mM Tris-HCl (pH 7.5), 1 mM EDTA, 2 M NaCl). Then DNA dissolved in 300 μl 10 mM Tris-HCl (pH 7.4) was added into the bead solution and incubated at RT for 15 min with rotation. The beads were then separated on a magnet and biotinylated DNA was bound to the streptavidin beads.

#### Sequencing library construction

The library preparation processes were performed with streptavidin beads as described for the in situ Hi-C protocol. Briefly, the ends of sheared DNA were repaired and the biotin from un-ligated ends was removed, adapters were added to the A-tailed DNA fragments, and PCR was performed with eight to ten cycles using Illumina primers. Finally, DNA size selection was performed with 0.65–0.8× volume of AMPure XP beads to make sure the DNA length distributes between 300 and 500 bp. The library was quantified with Qubit and sequenced using an Illumina sequencing platform.

### OCEAN-C data processing

OCEAN-C reads were mapped and filtered similarly to the situ Hi-C data. Briefly, clean reads were first trimmed to 36 bp and then mapped to genome hg19 with bowtie2, and reads with MAPQ less than 1 were discarded. If a read pair locates in the same restriction fragment (MboI), it was classified as dangling ends (inward), self-cycled (outward), or dumped pairs (same strand) and discarded. For the remaining read pairs that mapped to two different restriction fragments, if the distance between these two fragments was less than 1 kb, the read pairs were discarded due to the two ends’ close distance in sequence. The remaining read pairs were considered valid and used to call peaks and generate interaction heat maps. In the U266 cell line, the OCEAN-C peaks, which were defined as HOCIs, were determined by the ZINBA algorithm from the filtered data with the same parameters used for FAIRE-seq. In RPMI-8226 and GM12878 cell lines, HOCIs were called by ZINBA with the “pscl” method from the filtered data since the signals were too weak to be selected using the “mixture” method. HOCIs overlapping both H3K4me3 ChIP-seq peaks and gene promoters (2 kb up to genes’ transcription start sites) were defined as promoter HOCIs, and the rest which overlapping both H3k4me1 and H3K27Ac ChIP-seq peaks were defined as enhancer HOCIs. HOCI interactions, ChIA–PET interactions, gene densities, and gene transcription were examined using the WashU Epigenome Browser, and the network of HOCIs was constructed using the ggnet R package.

### Statistical analysis

The *p* values in Figs. 4e and 5b were generated using *t*-test with specified two-group data.

### Identification of super-enhancers

We used the rose software to identify enhancers [29]. First, enhancers were defined by H3K27ac ChIP-seq enriched regions. Second, the total background-subtracted ChIP-seq binding signals of DP1 or E2F1 were used to rank all enhancers and plotted (in units of rpm/bp). Finally, the x-axis points were identified where a line with slope of 1 was tangent to the curve, and the enhancers to the right of this point were defined as super-enhancers. Enhancers within 12.5 kb were stitched together, and regions within 2 kb of transcription start sites were considered as promoters rather than enhancers. The ChIP-seq data (H3K27ac, DP1 and E2F1) of U266 were downloaded from BipProject of NCBI (PRJNA319620).   

### Identification of broad H3K4me3 peaks

Broad H3K4me3 peaks were called from ChIP-seq data downloaded from ENCODE using MACS2 [38]. The “gapped peaks” were ranked by their width. The top 5% of peaks in width were defined as broad H3K4me3 peaks.

### Motif analysis

We use Homer [39] to find enrichment of sequence motifs. HOCIs and ChIA-PET anchors in bed format were used as input. The “Known Results” were used as final results.

### KEGG pathway enrichment analysis

We use DAVID [40] to find KEGG pathway enrichment. Genes whose promoters overlap with HOCIs were used as background, while genes whose promoters overlap with both HOCIs and broad H3K4me3 peaks were used as the input gene list.

## Additional files

Additional file 1: **Figures S1–S11.** (PDF 5443 kb)

Additional file 2: **Table S1.** Summary of OCEAN-C, FAIRE-seq, Hi-C, and RNA-seq data. (PDF 19 kb)

Additional file 3: **Table S2.** Summary of HOCI and gene expression data analysis. (PDF 22 kb)

Additional file 4: **Table S3.** Information of public data used in this study. (PDF 20 kb)
