# Supplementary material for: OCEAN-C: mapping hubs of open chromatin interactions across the genome reveals gene regulatory networks
Source: Genome Biol. 2018 Apr 24;19:54. doi: 10.1186/s13059-018-1430-4 (PMC5926533; doi:10.1186/s13059-018-1430-4)
Supplement: Supplementary file 1 — Figures S1–S11. (PDF 5443 kb) [file 13059_2018_1430_MOESM1_ESM.pdf]

Figure S1

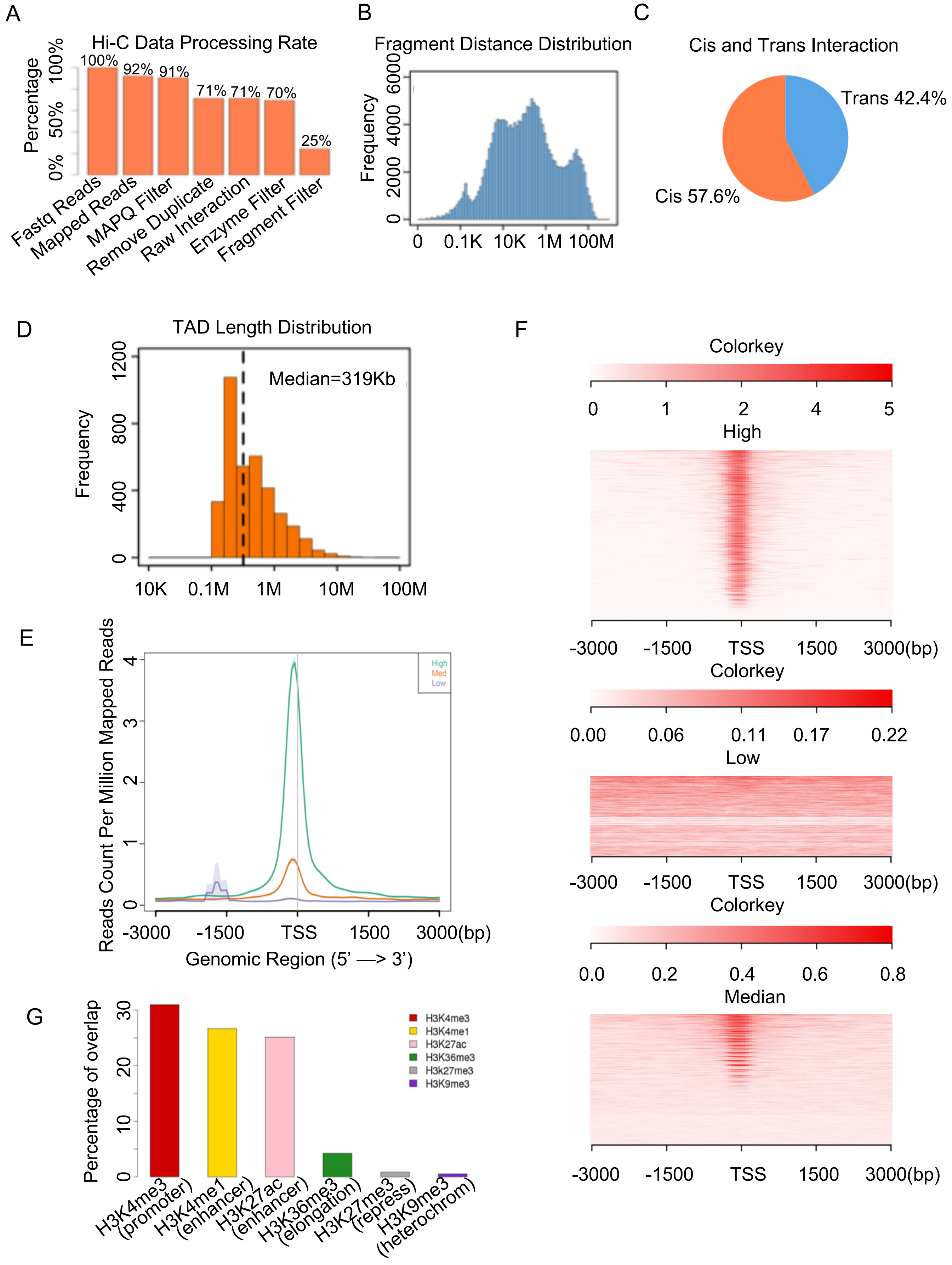
A
Hi-C Data Processing Rate
Percentage
0%
50%
100%
100%
92%
91%
71%
71%
70%
25%
Fastq Reads
Mapped Reads
MAPQ Filter
Remove Duplicate
Raw Interaction
Enzyme Filter
Fragment Filter
B
Fragment Distance Distribution
Frequency
0
2000
4000
6000
0
0.1K
10K
1M
100M
C
Cis and Trans Interaction
Trans 42.4%
Cis 57.6%
D
TAD Length Distribution
Median=319Kb
Frequency
0
400
800
1200
10K
0.1M
1M
10M
100M
E
Reads Count Per Million Mapped Reads
0
1
2
3
4
High
Med
Low
-3000
-1500
TSS
1500
3000(bp)
Genomic Region (5' —> 3')
F
Colorkey
0
1
2
4
5
High
-3000
-1500
TSS
1500
3000(bp)
Colorkey
0.00
0.06
0.11
0.17
0.22
Low
-3000
-1500
TSS
1500
3000(bp)
Colorkey
0.0
0.2
0.4
0.6
0.8
Median
-3000
-1500
TSS
1500
3000(bp)
G
Percentage of overlap
0
10
20
30
H3K4me3
H3K4me1
H3K27ac
H3K36me3
H3k27me3
H3K9me3
H3K4me3 (promoter)
H3K4me1 (enhancer)
H3K27ac (enhancer)
H3K36me3 (elongation)
H3K27me3 (repress)
H3K9me3 (heterochrom)

Supplementary Figure 1. Quality control of Hi-C and FAIRE-seq experiments. (A–D) Quality control of the Hi-C experiment (U266 cells). (A) Proportion of filtered reads in Hi-C data processing. (B) The distribution of fragment distances in cleaned Hi-C data. (C) Percentage of inter-chromosomal (trans) and intra-chromosomal (cis) interaction reads of clean Hi-C data. (D) The length distribution of topological associated domains (TADs). (E–G) Quality control of the FAIRE-seq experiment (U266 cells). (E) Reads distribution of FAIRE-seq peaks around transcription start sites (TSSs). The peak signals are averaged for three groups according to corresponding RNA-seq expression: high (top 30%), median (middle 40%), and low (bottom 30%). (F) Heat map of FAIRE-seq peaks around TSSs ranked by gene expression. (G) Percent of FAIRE-seq peaks overlapping various histone modification markers.

Figure S2

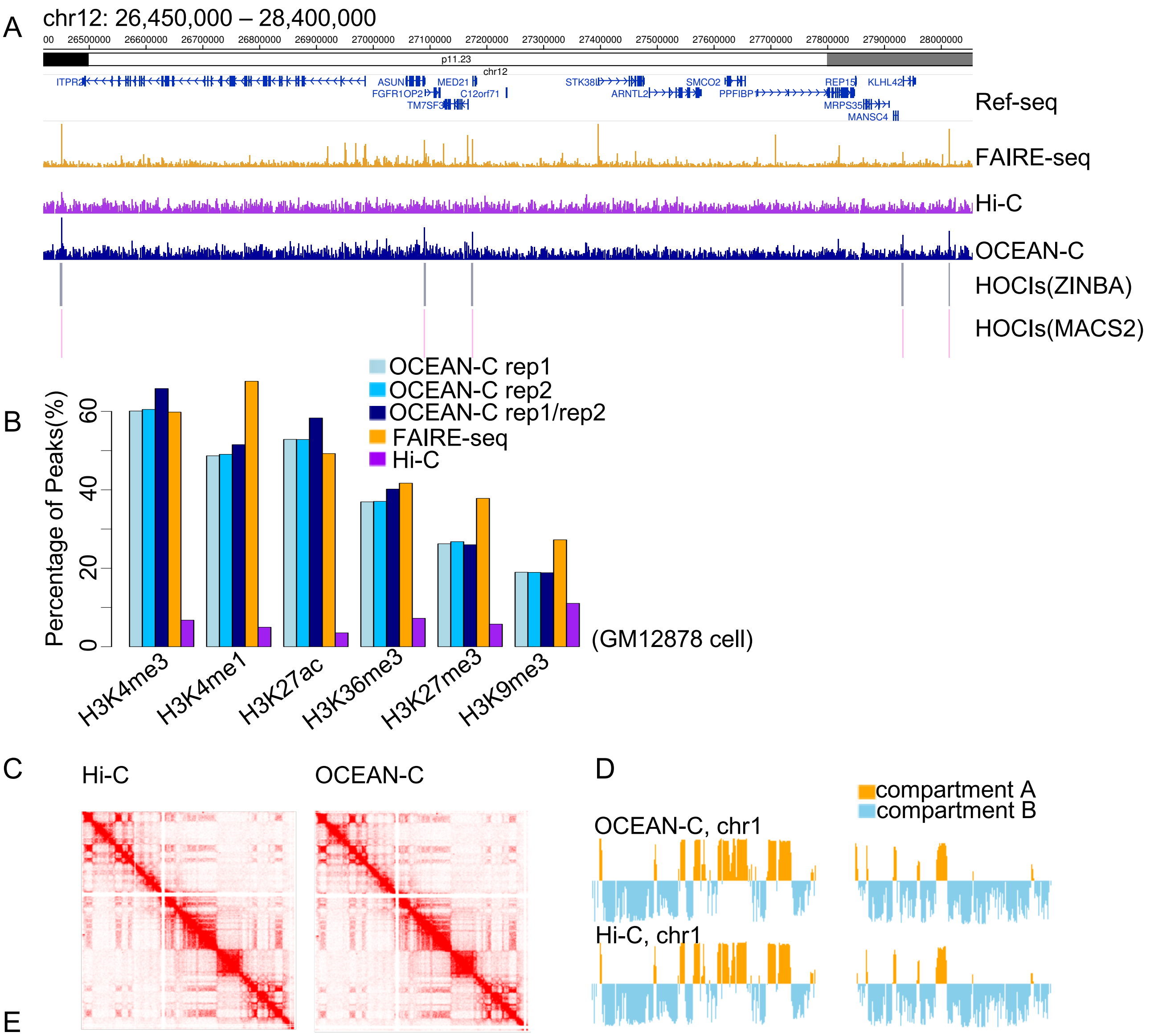

E

	chr1	chr2	chr3	chr4	chr5	chr6	chr7	chr8
cor(ICE)	0.9916	0.9883	0.9862	0.9874	0.9885	0.9856	0.9963	0.9930
cor(raw)	0.9934	0.9928	0.9942	0.9941	0.9924	0.9922	0.9949	0.9933

	chr9	chr10	chr11	chr12	chr13	chr14	chr15	chr16
cor(ICE)	0.9837	0.9857	0.9906	0.9833	0.9763	0.9894	0.9985	0.9870
cor(raw)	0.9943	0.9932	0.9919	0.9926	0.9942	0.9924	0.9950	0.9917

	chr17	chr18	chr19	chr20	chr21	chr22	chrX	chrY
cor(ICE)	0.9929	0.9985	0.9989	0.9881	0.9987	0.9989	0.9850	NA
cor(raw)	0.9923	0.9933	0.9904	0.9925	0.9943	0.9928	0.9928	NA

F

	U266	RPMI8226	GM12878 Rep1	GM12878 Rep2
OCEAN-C / Hi-C conserved compartment	95.6%	91.0%	91.2%	93.3%
OCEAN-C / Hi-C conserved TAD	92.3%	94.1%	93.3%	93.2%

Supplementary Figure 2. Comparisons between OCEAN-C and Hi-C data. (A) The browser view of a 2 Mb genomic region showing HOCIs called with ZINBA and MACS2 algorithms, as well as the read depth of Hi-C, OCEAN-C and FAIRE-seq data in U266 cells. (B) Overlaps between histone modification markers and the peaks determined by OCEAN-C, FAIRE-seq and Hi-C in GM12878 cells. (C) Interaction heat map of chromosome X from Hi-C and OCEAN-C data (U266). (D) Compartment A/B of chromosome 1 determined by Hi-C and OCEAN-C (U266). (E) Pearson correlations of raw or ICE normalized Hi-C and OCEAN-C interaction matrices of each chromosome (U266). The upper triangular interaction matrices (including diagonal) are used to compute correlations. (F) Proportions of conserved compartments and TADs between Hi-C and OCEAN-C data. For conserved compartments, the proportion of 200 kb bins with the same compartment types between Hi-C and OCEAN-C is computed. When two TADs called from Hi-C and OCEAN-C data overlap with each other by >70% in length, they are regarded as conserved TADs.

Figure S3

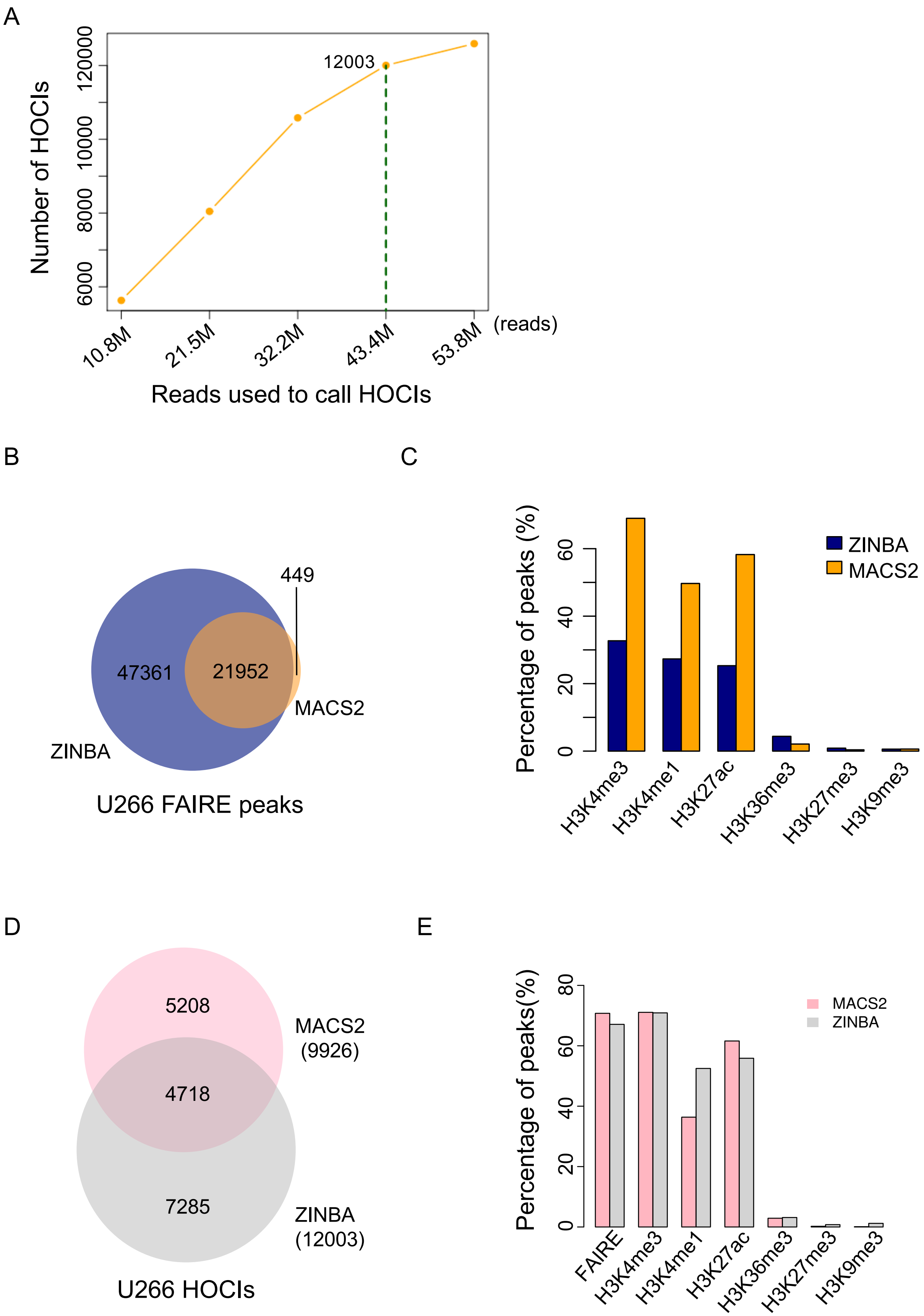

A
Number of HOCIs
6000
8000
10000
120000
12003
10.8M
21.5M
32.2M
43.4M
53.8M
(reads)
Reads used to call HOCIs
B
449
47361
21952
MACS2
ZINBA
U266 FAIRE peaks
C
Percentage of peaks (%)
0
20
40
60
ZINBA
MACS2
H3K4me3
H3K4me1
H3K27ac
H3K36me3
H3K27me3
H3K9me3
D
5208
4718
7285
MACS2
(9926)
ZINBA
(12003)
U266 HOCIs
E
Percentage of peaks(%)
0
20
40
60
80
MACS2
ZINBA
FAIRE
H3K4me3
H3K4me1
H3K27ac
H3K36me3
H3K27me3
H3K9me3

Supplementary Figure 3. Comparisons between two peak calling algorithms. (A) HOCIs called with different sequencing depth of U266 cells. M: million reads. (B) Venn diagram of the open chromatin peaks called from FAIRE-seq data using ZINBA and MACS2 algorithms (U266). (C) Percentage of open chromatin peaks called from FAIRE-seq data using ZINBA and MACS2 that overlap with different histone modification markers (U266). (D) Venn diagram of the HOCIs called from OCEAN-C data using ZINBA and MACS2 (U266). (E) Percentage of HOCIs called from OCEAN-C data using ZINBA and MACS2 that overlap with different histone modification markers (U266).

Figure S4

A

DNase-C Cleaned-Pairs	DNase-C Cis Cleaned-Pairs	DNase-C Peaks	DNase-seq Peaks	Overlapped Peaks
72769564	48608321	5958	17883	18

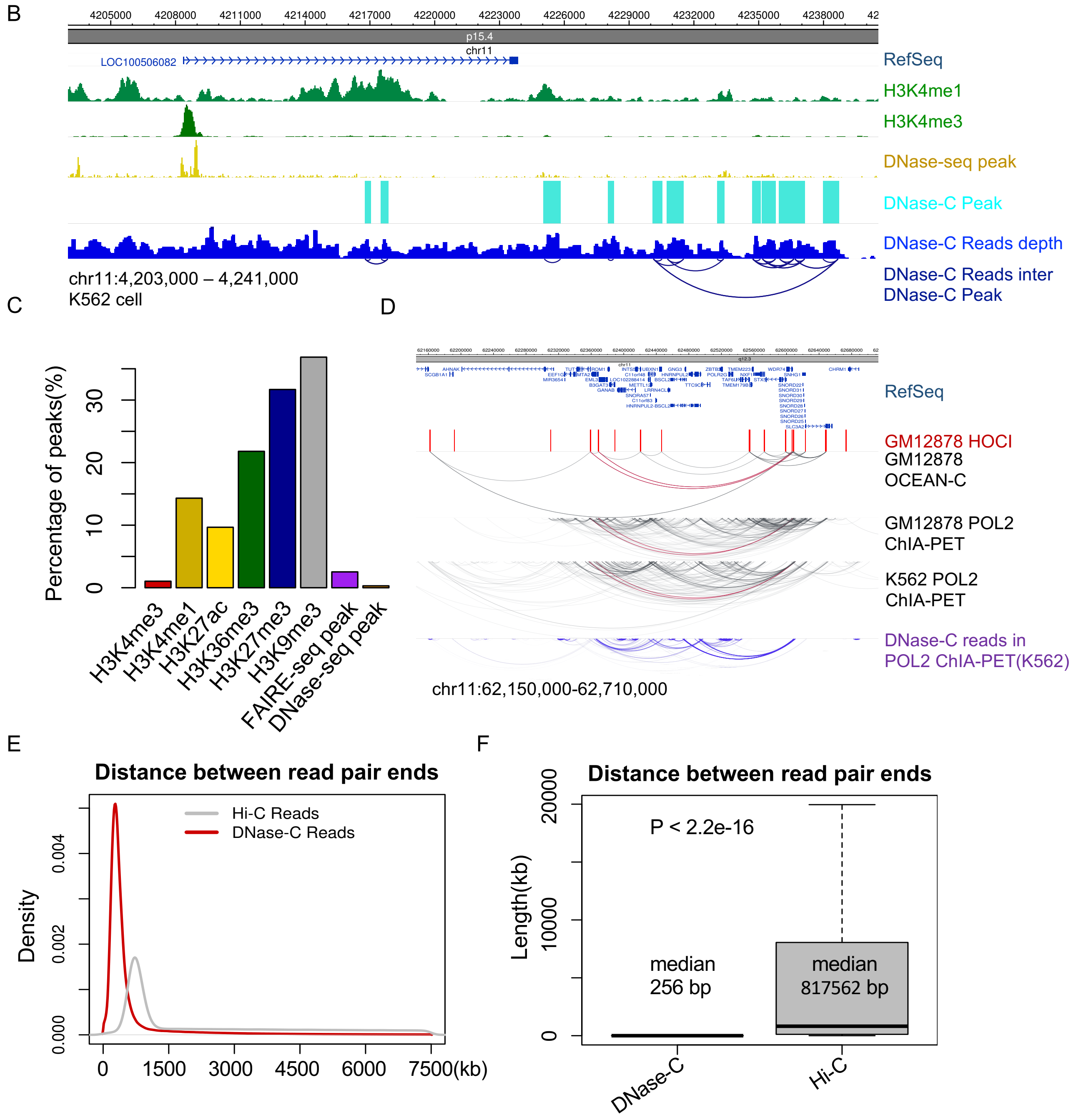

Supplementary Figure 4. Comparisons between DNase-C and OCEAN-C. (A) Statistics of DNase-seq and DNase-C peak calling steps (K562 cells). (B) The browser view of a 38 kb genomic region showing DNase-C read pairs connecting DNase-C peaks, together with the signals of DNase I and histone modification markers H3K4me1and H3K4me3. (C) Barplot showing the overlaps between DNase-C peaks and open chromatin related markers. (D) The browser view of a 560 kb genomic region showing read pairs of DNase-C, POL2 ChIA-PET and OCEAN-C data. OCEAN-C: read pairs with both ends mapped to HOCIs, 'DNase-C reads in POL2 ChIA-PET': read pairs from DNase-C with both ends mapped to POL2 ChIA-PET cluster anchors. (E) The distribution of distance between two ends of read pairs from Hi-C and DNase-C. (F) Boxplots showing the distance between two ends of read pairs from Hi-C and DNase-C. p-value is generated using Wilcoxon test.

Figure S5

A

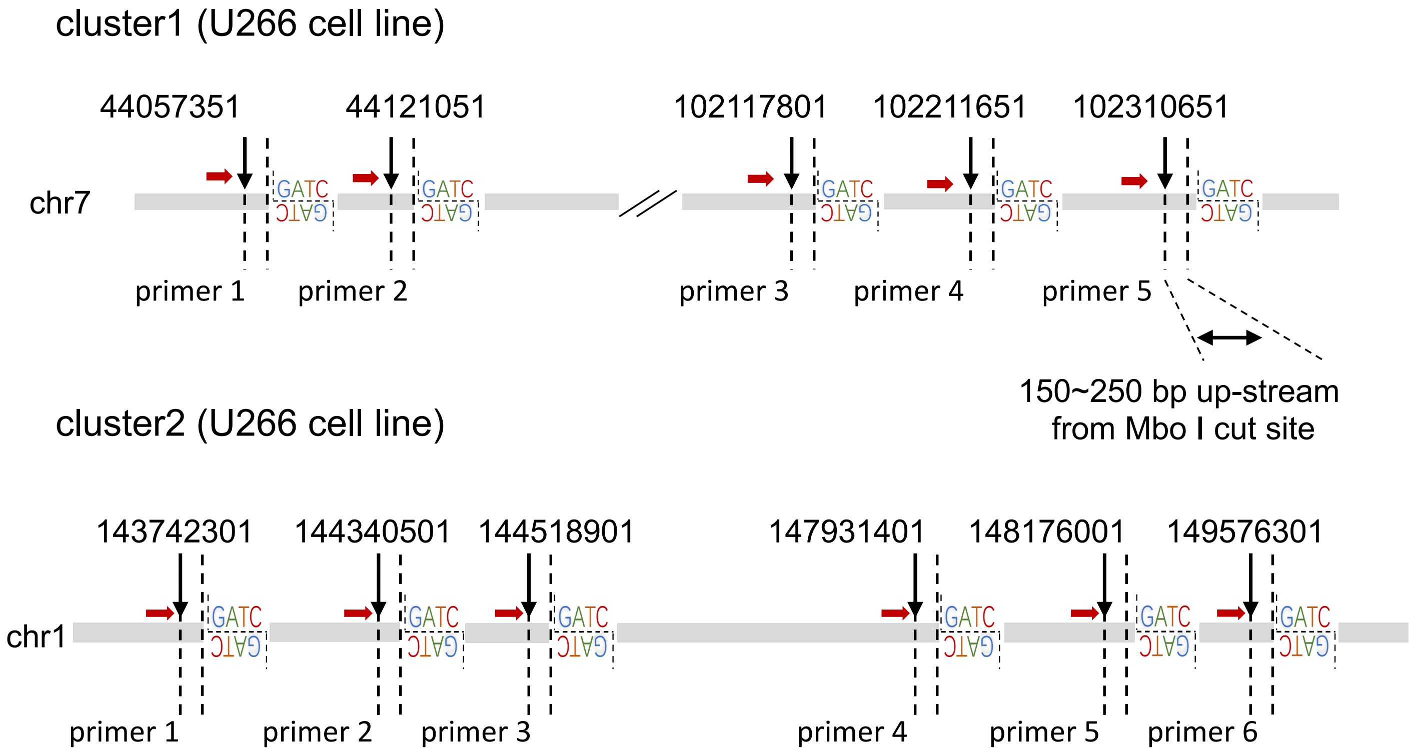
cluster1 (U266 cell line)
44057351
44121051
102117801
102211651
102310651
chr7
GATC
primer 1
primer 2
primer 3
primer 4
primer 5
150~250 bp up-stream
from Mbo I cut site
cluster2 (U266 cell line)
143742301
144340501
144518901
147931401
148176001
149576301
chr1
primer 1
primer 2
primer 3
primer 4
primer 5
primer 6

B

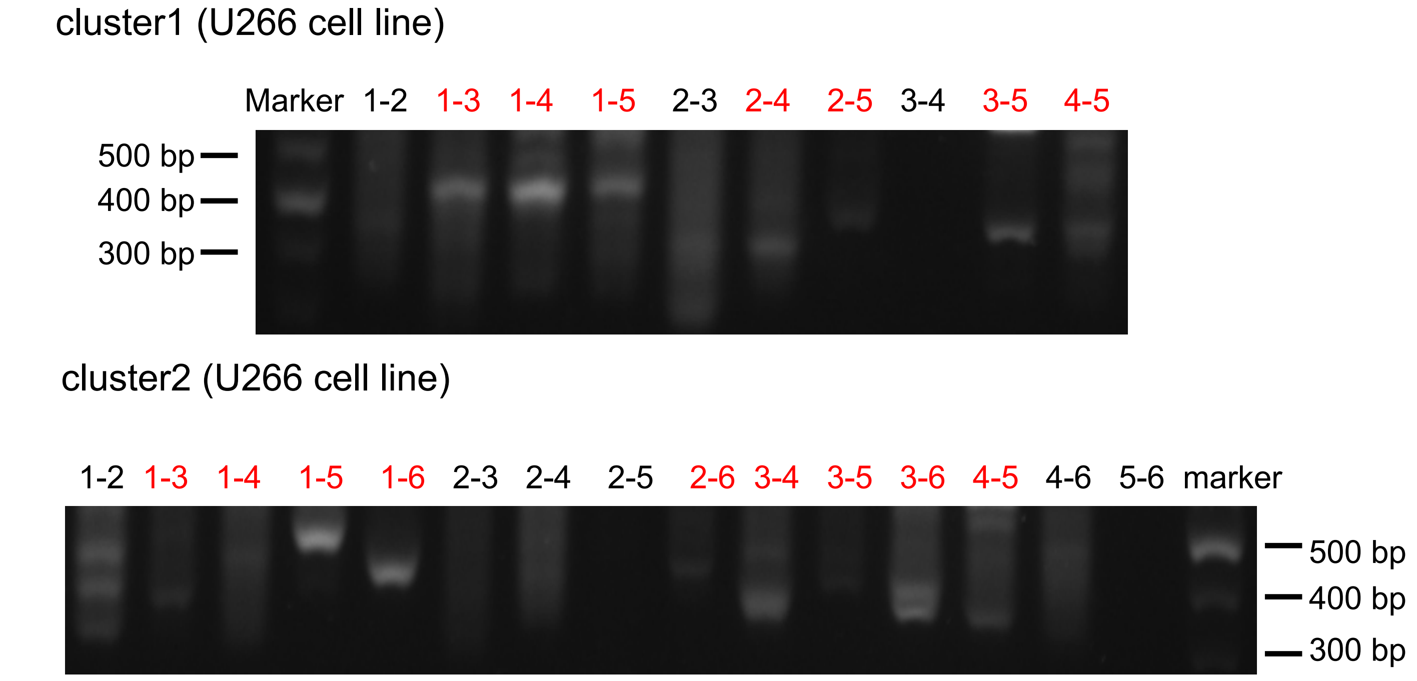
cluster1 (U266 cell line)
Marker 1-2 1-3 1-4 1-5 2-3 2-4 2-5 3-4 3-5 4-5
500 bp
400 bp
300 bp
cluster2 (U266 cell line)
1-2 1-3 1-4 1-5 1-6 2-3 2-4 2-5 2-6 3-4 3-5 3-6 4-5 4-6 5-6 marker
500 bp
400 bp
300 bp

Supplementary Figure 5. 3C validation of chromatin interaction clusters captured by OCEAN-C. (A) Schematic plot of the sites of 3C primers in two interaction clusters. 'GATC' is the cutting site of MboI. Red arrows: 3C primer sites; vertical dashed lines: 150~250 bp upstream of the MboI cutting sites; number on the top: 5' genome positions of the 3C primers. (B) PCR results of primer combinations from (A). Red labels indicate detected 3C interactions between indicated primer pairs, while black labels indicate failed detection.

Figure S6

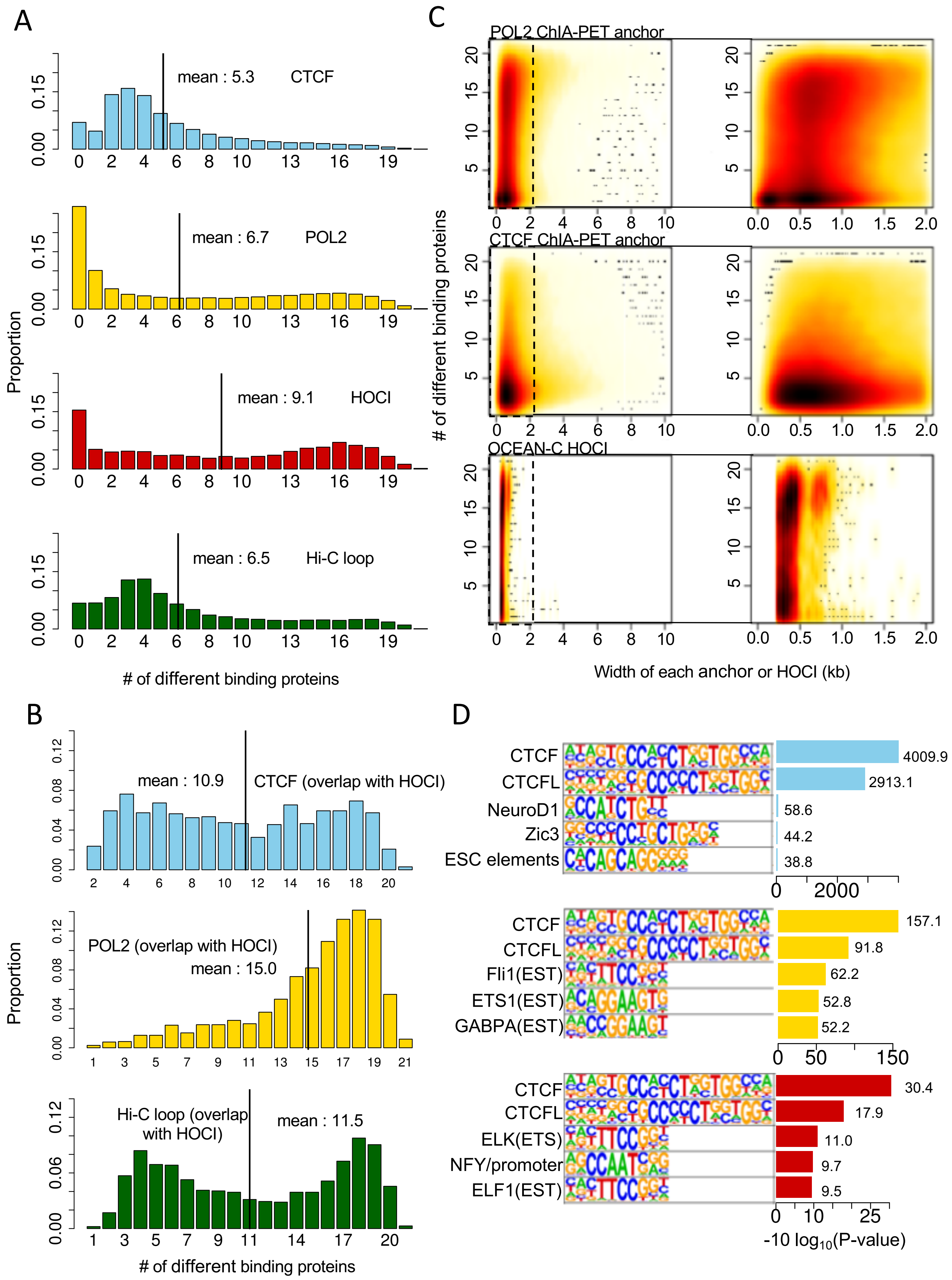
A
mean : 5.3 CTCF
mean : 6.7 POL2
mean : 9.1 HOCI
mean : 6.5 Hi-C loop
Proportion
# of different binding proteins
C
POL2 ChIA-PET anchor
CTCF ChIA-PET anchor
OCEAN-C HOCI
# of different binding proteins
Width of each anchor or HOCI (kb)
B
mean : 10.9
CTCF (overlap with HOCI)
POL2 (overlap with HOCI)
mean : 15.0
Hi-C loop (overlap with HOCI)
mean : 11.5
Proportion
# of different binding proteins
D
CTCF 4009.9
CTCFL 2913.1
NeuroD1 58.6
Zic3 44.2
ESC elements 38.8
CTCF 157.1
CTCFL 91.8
Fli1(EST) 62.2
ETS1(EST) 52.8
GABPA(EST) 52.2
CTCF 30.4
CTCFL 17.9
ELK(ETS) 11.0
NFY/promoter 9.7
ELF1(EST) 9.5
-10 log10(P-value)

Supplementary Figure 6. Characteristics of HOCIs. (A) Distribution of the number of different DNA-binding proteins for HOCIs, ChIA-PET anchors and Hi-C loop anchors (GM12878). (B) Distribution of the number of DNA-binding proteins for ChIA-PET anchors and Hi-C loop anchors that overlap with HOCIs (GM12878). (C) Contour plot showing the relationship between the width and the number of DNA-binding proteins for HOCIs and ChIA-PET anchors. The areas outlined by dashed rectangles in the left panels are enlarged in the right panels. (D) Top 5 enriched motif families in CTCF ChIA-PET anchors (blue), POL2 ChIA-PET anchors (gold) and HOCIs (red).

Figure S7

A

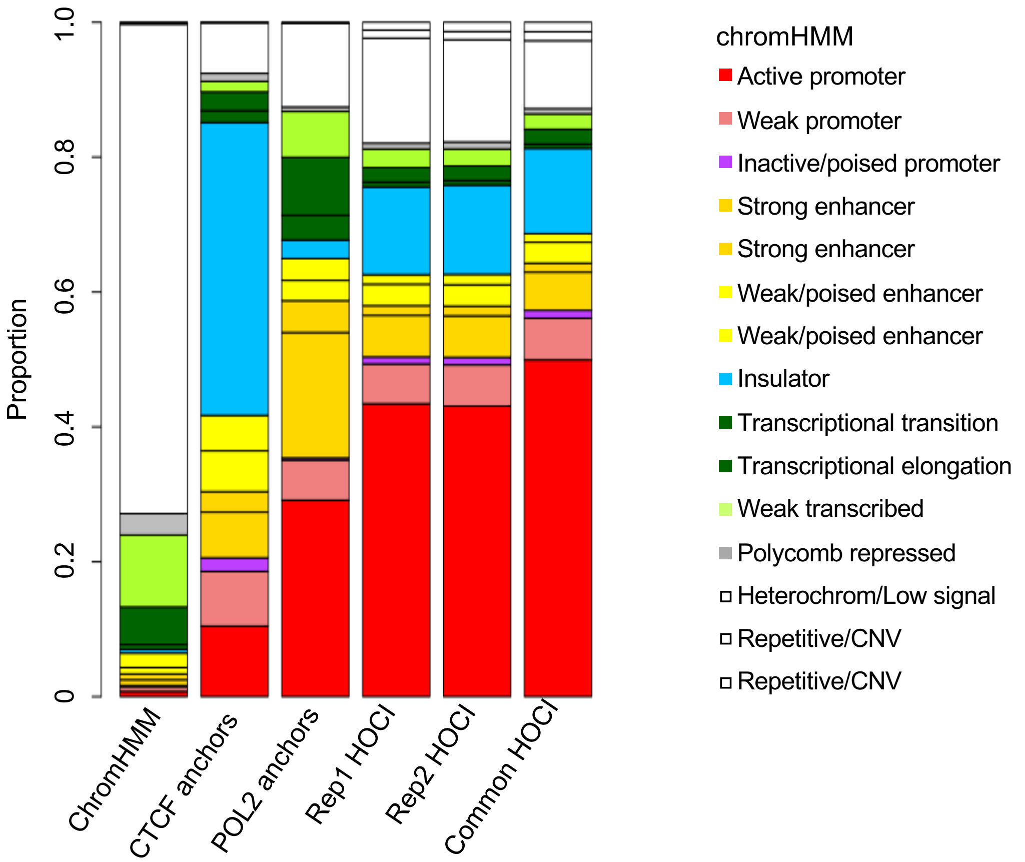

Proportion
0
0.2
0.4
0.6
0.8
1.0
ChromHMM
CTCF anchors
POL2 anchors
Rep1 HOCI
Rep2 HOCI
Common HOCI
chromHMM
Active promoter
Weak promoter
Inactive/poised promoter
Strong enhancer
Strong enhancer
Weak/poised enhancer
Weak/poised enhancer
Insulator
Transcriptional transition
Transcriptional elongation
Weak transcribed
Polycomb repressed
Heterochrom/Low signal
Repetitive/CNV
Repetitive/CNV

B

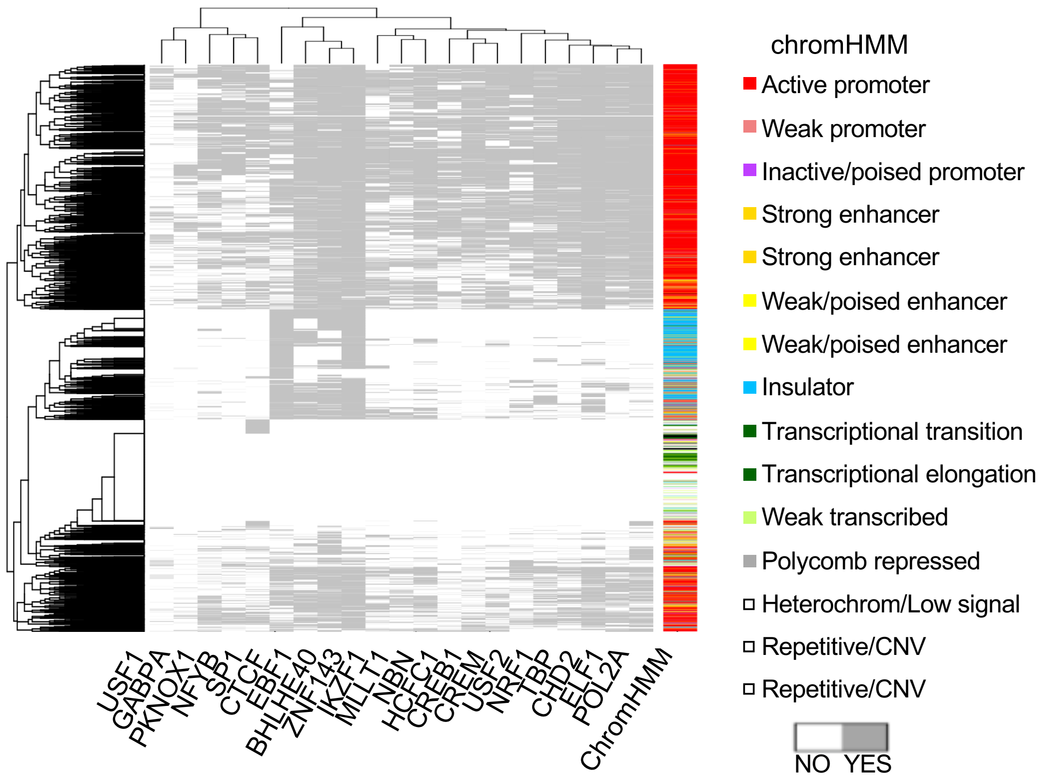

USF1
GABPA
PKNOX1
NFYB
SP1
CTCF
EBF1
BHLHE40
ZNF143
IKZF1
MLLT1
NBN
HCFC1
CREB1
CREM
USF2
NRF1
TBP
CHD2
ELF1
POL2A
ChromHMM
chromHMM
Active promoter
Weak promoter
Inactive/poised promoter
Strong enhancer
Strong enhancer
Weak/poised enhancer
Weak/poised enhancer
Insulator
Transcriptional transition
Transcriptional elongation
Weak transcribed
Polycomb repressed
Heterochrom/Low signal
Repetitive/CNV
Repetitive/CNV
NO
YES

Supplementary Figure 7. Genomic properties of HOCIs (A) Chromatin state distributions of ChIA-PET anchors and HOCIs (GM12878). (B) Clustering of HOCIs according to the binding status of 21 DNA binding proteins.

Figure S8

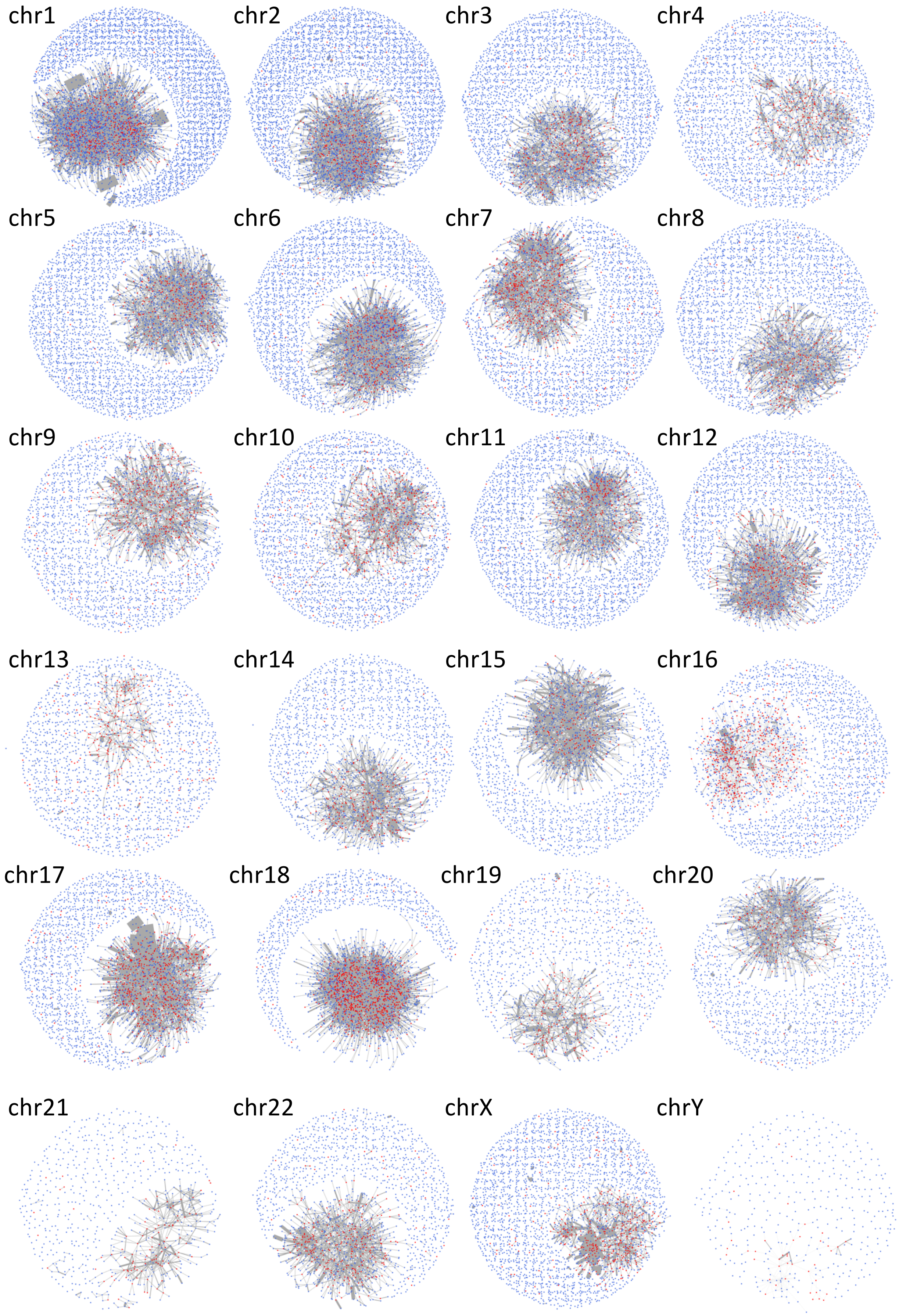
chr1
chr2
chr3
chr4
chr5
chr6
chr7
chr8
chr9
chr10
chr11
chr12
chr13
chr14
chr15
chr16
chr17
chr18
chr19
chr20
chr21
chr22
chrX
chrY

Supplementary Figure 8. Interaction network of HOCIs in individual chromosomes. Blue : open chromatin peaks identified by FAIRE-seq, red: open chromatin regions that overlap with HOCIs. The thickness of edges indicates the interaction intensity between HOCIs.

Figure S9

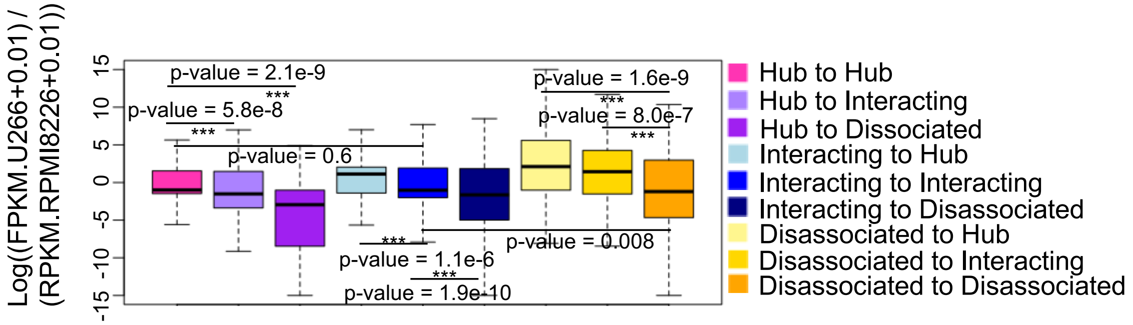

Supplementary Figure 9. The changes of gene transcription are consistent with changes of HOCI-related interactions. Boxplots show the differentially expression of genes classified by changes of HOCI-related interactions. p-values are generated using *t*-test.
