# Supplementary material for: OCEAN-C: mapping hubs of open chromatin interactions across the genome reveals gene regulatory networks
Source: Genome Biol. 2018 Apr 24;19:54. doi: 10.1186/s13059-018-1430-4 (PMC5926533; doi:10.1186/s13059-018-1430-4)
Supplement: Supplementary file 2 — Table S1. Summary of OCEAN-C, FAIRE-seq, Hi-C, and RNA-seq data. (PDF 19 kb) [file 13059_2018_1430_MOESM2_ESM.pdf]

**Table S1. Summary of OCEAN-C, FAIRE-seq, Hi-C and RNA-seq data.**

A. Summary of OCEAN-C Data

| Cell line | Replicate | Sequencing reads | Mapped and dup removed reads | Cis interact read pairs | HOCI |
|---|---|---|---|---|---|
| **U266** | Replicate 1 | 368411842 | 266032488 | 53841187 | 12003 |
| **RPMI8226** | Replicate 1 | 673360694 | 394655862 | 103416714 | 10686 |
| **GM12878** | Replicate 1 | 407593898 | 291014471 | 64334977 | 9505 |
| **GM12878** | Replicate 2 | 414533876 | 290404915 | 73268567 | 9787 |
| **GM12878** | Combined | 822127774 | 581419386 | 137603544 | 12231 |

B. Summary of FAIRE-seq Library Data

| Cell line | Replicate | Sequencing reads | Mapped and filtered reads | Open chromatin |
|---|---|---|---|---|
| **U266** | Replicate 1 | 235007648 | 35593971 | 69313 |
| **RPMI8226** | Replicate 1 | 226477078 | 29727633 | 92882 |
| **GM12878** | Replicate 1 | 200098720 | 63104334 | 48556 |

C. Summary of Hi-C Library Data

| Cell line | Replicate | Sequencing reads | Mapped and dup removed reads | Cis interact read pairs |
|---|---|---|---|---|
| **U266** | Replicate 1 | 208522886 | 183274599 | 43383922 |
| **RPMI8226** | Replicate 1 | 244454180 | 211240383 | 48657005 |
| **GM12878** | Replicate 1 | 409620210 | 234606571 | 50428576 |

D. Summary of RNA-seq Library Data

| Cell line | Replicate | Sequencing reads | Mapped and filtered reads |
|---|---|---|---|
| **U266** | Replicate 1 | 32184358 | 30581367 |
| **U266** | Replicate 2 | 40382044 | 37450514 |
| **U266** | Replicate 3 | 42281606 | 40679308 |
| **U266** | Combined | 114848008 | 108711189 |
| **RPMI8226** | Replicate 1 | 34837134 | 32559191 |
| **RPMI8226** | Replicate 2 | 40040036 | 37868327 |
| **RPMI8226** | Replicate 3 | 37013790 | 35137569 |
| **RPMI8226** | Combined | 111890960 | 105565087 |
