# Supplementary material for: OCEAN-C: mapping hubs of open chromatin interactions across the genome reveals gene regulatory networks
Source: Genome Biol. 2018 Apr 24;19:54. doi: 10.1186/s13059-018-1430-4 (PMC5926533; doi:10.1186/s13059-018-1430-4)
Supplement: Supplementary file 3 — Table S2. Summary of HOCI and gene expression data analysis. (PDF 22 kb) [file 13059_2018_1430_MOESM3_ESM.pdf]

Table S2. Summary of HOCI and gene expression data analysis

A. Comparison of HOCI, ChIA-PET anchor and Hi-C loop anchor

	Overlapped HOCIs (total: 12231)
Anchors of CTCF ChIA-PET(185614)	4998(41%)
Anchors of Pol II ChIA-PET(227182)	4454(47%)
Loop anchors of in situ Hi-C(12903)	2524(21%)

B. Comparison of TAD boundary detected from Hi-C data and HOCI detected from OCEAN-C data

	Total HOCIs	HOCIs overlapped with TAD boundaries	Number of Boundaries	mean of random sampling HOCIs overlapped with TAD boundaries	P-value
**U266**	12003	1152	4253	709	5.2e-35
**RMPI8226**	14592	898	4429	617	1.0e-28
**GM12878 rep1**	9505	782	4259	500	2.3e-34
**GM12878 rep2**	9787	802	4259	515	5.3e-25

C. RPKM of three different types of genes

Types	U266			RPMI8226		
	Gene Number (All:22372)	Exp Gene (RPKM>= 0.5)	Mean (RPKM)	Gene Number (All:22372)	Exp Gene (RPKM>= 0.5)	Mean (RPKM)
**Hub**	5780	5072(87.8%)	46.8	4206	3724(88.5%)	48.2
**Interacting**	10617	5230 (49.3%)	42.7	13401	6838(51.0%)	43.1
**Dissociative**	5975	1301(21.8%)	18.5	4765	1187(24.9%)	22.0

D. The components of expressed gene in U266 and RPMI8226 cell line

	U266	RPMI8226
**Expressed Genes (RPKM >= 0.5)**	11603	11749
**Expressed Hub Genes**	5072 (43.7%)	3724 (31.7%)
**Expressed Interacting Genes**	5230 (45%)	6838(58.2%)
**Expressed Hub/Interacting Gene**	10302(88.7%)	10562(89.9%)

E. The components of housekeeping gene

	Types	U266 cell	RPMI8226 cell
**Housekeeping Gene (3640)**	**Hub**	1901(52.2%)	1415(38.9%)
	**Interacting**	1435(39.4%)	1971(54.1%)
	**Dissociative**	304(8.4%)	254(7%)
