# Supplementary material for: OCEAN-C: mapping hubs of open chromatin interactions across the genome reveals gene regulatory networks
Source: Genome Biol. 2018 Apr 24;19:54. doi: 10.1186/s13059-018-1430-4 (PMC5926533; doi:10.1186/s13059-018-1430-4)
Supplement: Supplementary file 4 — Table S3. Information of public data used in this study. (PDF 20 kb) [file 13059_2018_1430_MOESM4_ESM.pdf]

Table S3. Information of public data used in this study

A. ChIP-seq data of GM12878 cell line.

protein	ENCODE Accession	protein	ENCODE Accession
PKNOX1	ENCFF869BJB	USF1	ENCFF281OFW
BHLHE40	ENCFF431VZN	EBF1	ENCFF000VUS
ZNF143	ENCFF000WGJ	SP1	ENCFF000OEL
CREB1	ENCFF000NTW	NFYB	ENCFF000VZX
ELF1	ENCFF000NUX	HCFC1	ENCFF370WJQ
CHD2	ENCFF000VTS	IKZF1	ENCFF648SPM
GABPA	ENCFF249FIH	USF2	ENCFF000WFU
CREM	ENCFF500GPO	NRF1	ENCFF000WAH
TBP	ENCFF000WFG	CTCF	ENCFF389RQC
MLLT1	ENCFF076TDR	H3K4me3	ENCSR000AKA
POL2A	ENCFF865BUP	H3K27ac	ENCSR000AKC
NBN	ENCFF282JRK	H3K27me3	ENCSR000AKD
H3K4me1	ENCSR000AKF	H3K9me3	ENCSR000AOX
H3K36me3	ENCSR000AKE		

B. ChIA-PET and Hi-C loop data of GM12878 cell line

sample	GEO accession
CTCF ChIA-PET	GSM1872886
POL2 ChIA-PET	GSM1872887
Hi-C loop	GSE63525

C. ChIP-seq data of U266 cell lines

protein	U266 (SRA Accession)	protein	U266 (SRA Accession)
H3K4me3	ERR324261	H3K36me3	ERR324288
H3K4me1	ERR324265	H3K27me3	ERR324301
H3K27ac	ERR324308	H3K9me3	ERR324281
DP1	SRR3440074	E2F1	SRR3440075

D. Public data of K562 cell line

sample	GEO accession	sample	GEO accession
DNase-C	GSM1370434	FAIRE-seq	GSM864340
DNase-seq	GSM2400523	H3K9me3	GSM733776
POL2 ChIA-PET	GSM970213	H3K27me3	GSM733658
CTCF ChIA-PET	GSM970216	H3K27ac	GSM733656
H3K4me1	GSM733692	H3K4me3	GSM2534288
H3K36me3	GSM733714		
